# Supplementary figures and images for: The role of nuclear organization in trans-splicing based expression of heat shock protein 90 in Giardia lamblia
Source: PLoS Negl Trop Dis. 2021 Sep 24;15(9):e0009810. doi: 10.1371/journal.pntd.0009810 (PMC8494341; doi:10.1371/journal.pntd.0009810)

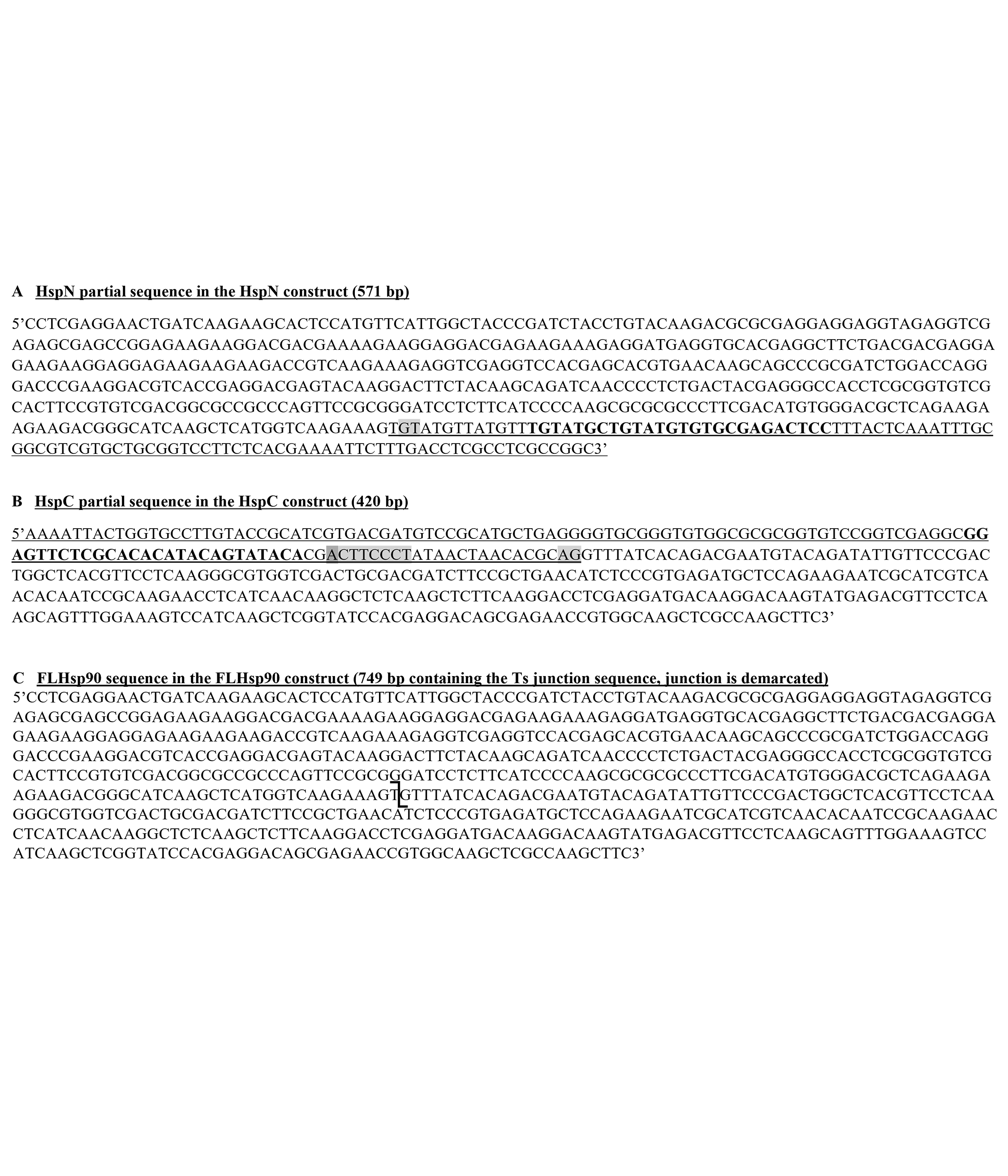

Supplement: S1 Fig — A, B) Underlined residues indicate the nucleotides spliced out after in vitro transcription and incubation in trans-splicing (ts) buffer. Nucleotides in bold are the complementary sequence elements in both the partial sequences resulting in pre-mRNAs harbouring complementary nucleotides after in vitro transcription. The nucleotides highlighted in the sequences represent the critical nucleotides, 5’ SS-GT in HspN clone (GU in HspN pre-mRNA); 3’SS-AG, polypyrimidine tract CCTCCCT (CCUCCCU in HspC pre-mRNA) and branch point Adenine in HspC clone, C) Sequence of FLHsp90 with the trans-spliced junction is demarcated. FLHsp90 clone after in vitro transcription would generate FLRNA of 947 nts which served as positive control for in vitro trans-splicing assay. (TIF) [file pntd.0009810.s001.tif]

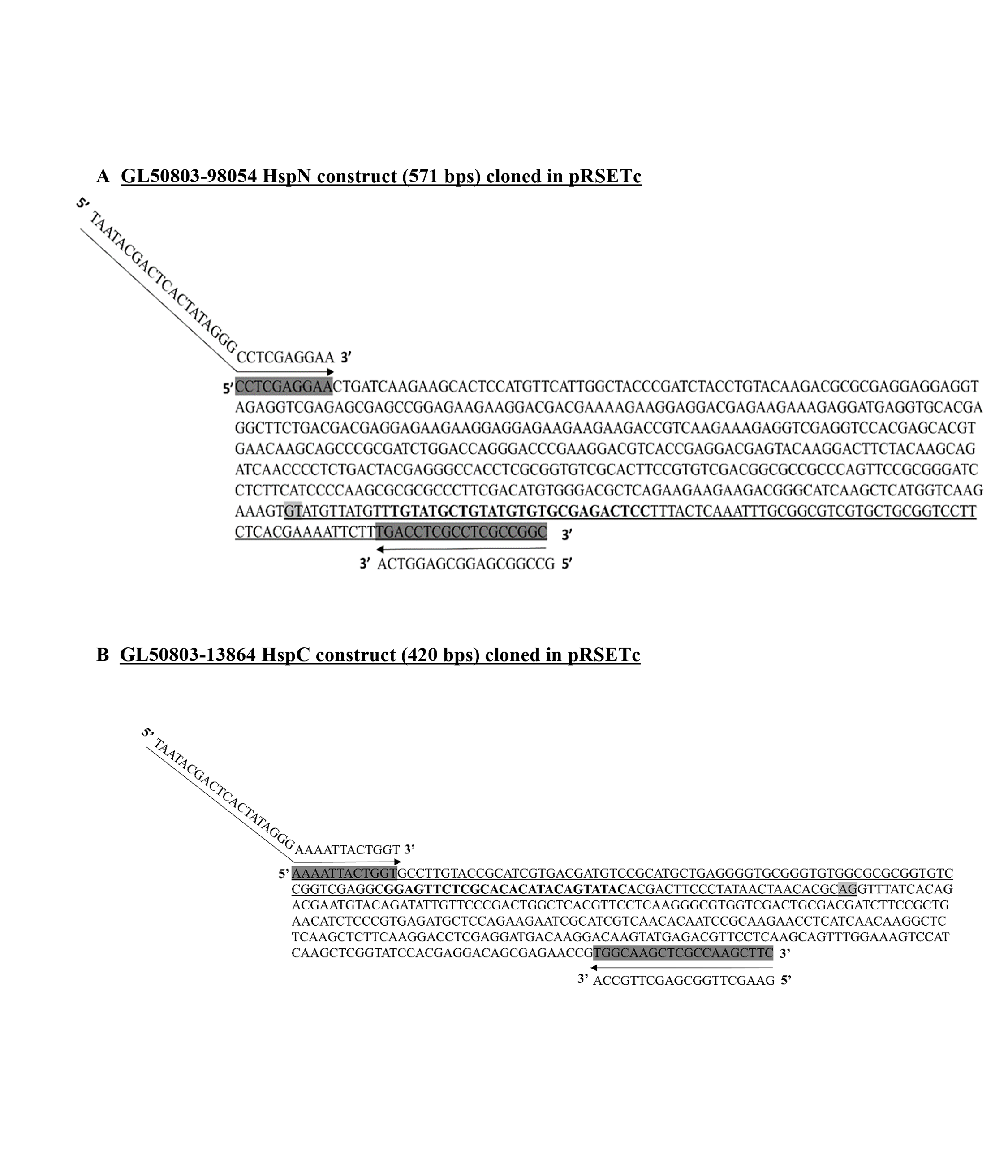

Supplement: S2 Fig — Underlined residues indicate the intronic regions of pre-mRNAs. Nucleotides in bold are the complementary sequence elements. The nucleotides highlighted in the sequences represent the critical nucleotides; splice sites 5’SS-GT and 3’SS-AG. Also, sequences highlighted at the beginning and at the end indicate forward and reverse primers used to generate corresponding pre-mRNAs lacking the MCS. Forward primer in each case begins with T7 promoter sequence (5’-TAATACGACTCACTATAGGG-3’) with 3 additional Gs at the 3’ end before the target specific sequence. S2A and S2B Fig indicate precisely the Primer design strategy for HspN and HspC pre-mRNAs to be employed for body labelling of the pre-mRNAs with biotin-16-UTP for the in-vitro RNA-protein pull down assay. Same primer sets were used to generate HspNΔ26 and HspCΔ27 from clones previously cloned lacking the complementary sequences [23]. (TIF) [file pntd.0009810.s002.tif]

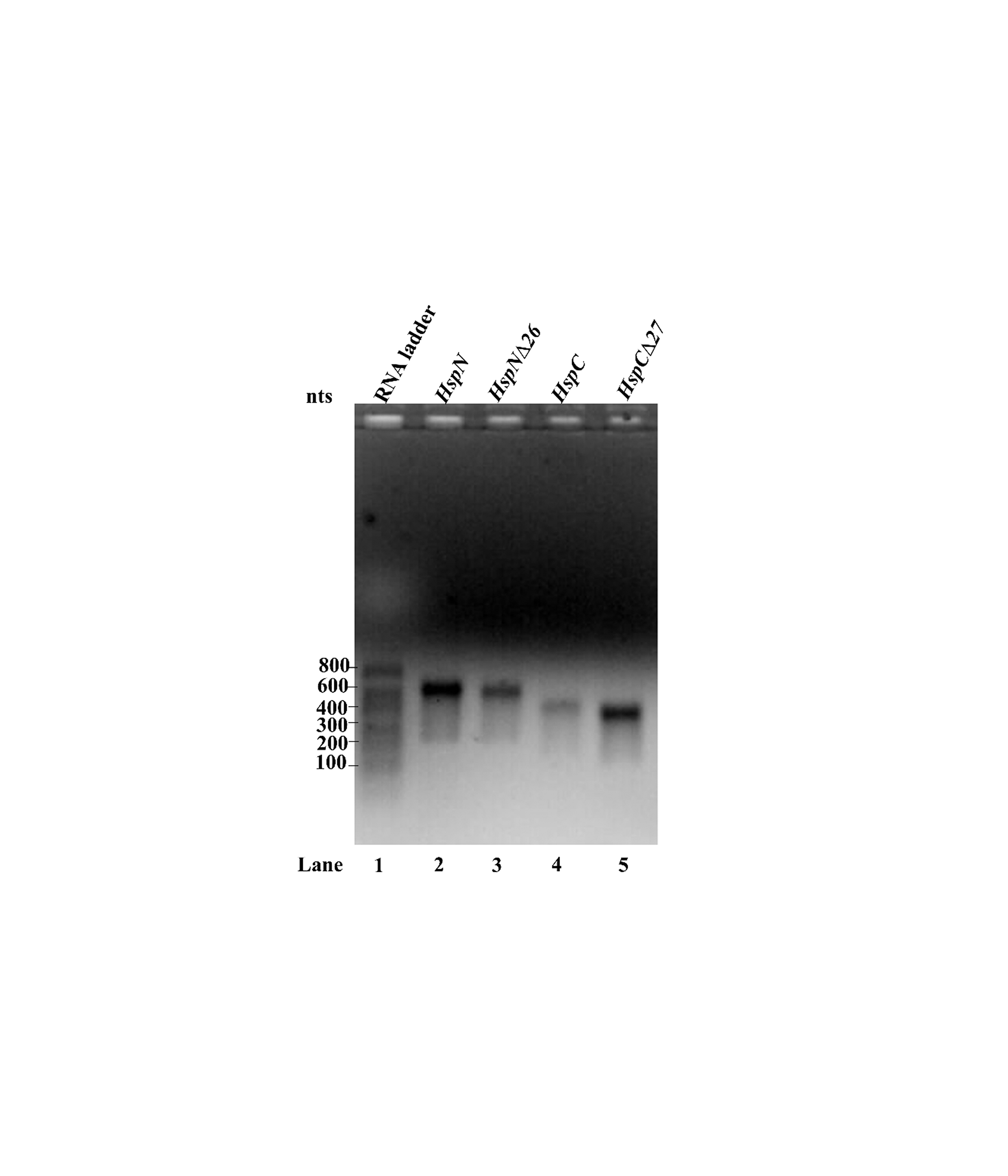

Supplement: S3 Fig — Linearized wild type and mutant plasmids were subjected to PCR using primer having T7 promoter sequence, the amplified product was gel purified and subjected to in vitro transcription and resolved on MOPS-formaldehyde agarose gel to check the integrity of the RNAs. All of the pre-mRNAs, mutant and wild type, were intact with a single compact band running at the corresponding sizes and hence were used for pull-down assay. (TIF) [file pntd.0009810.s003.tif]

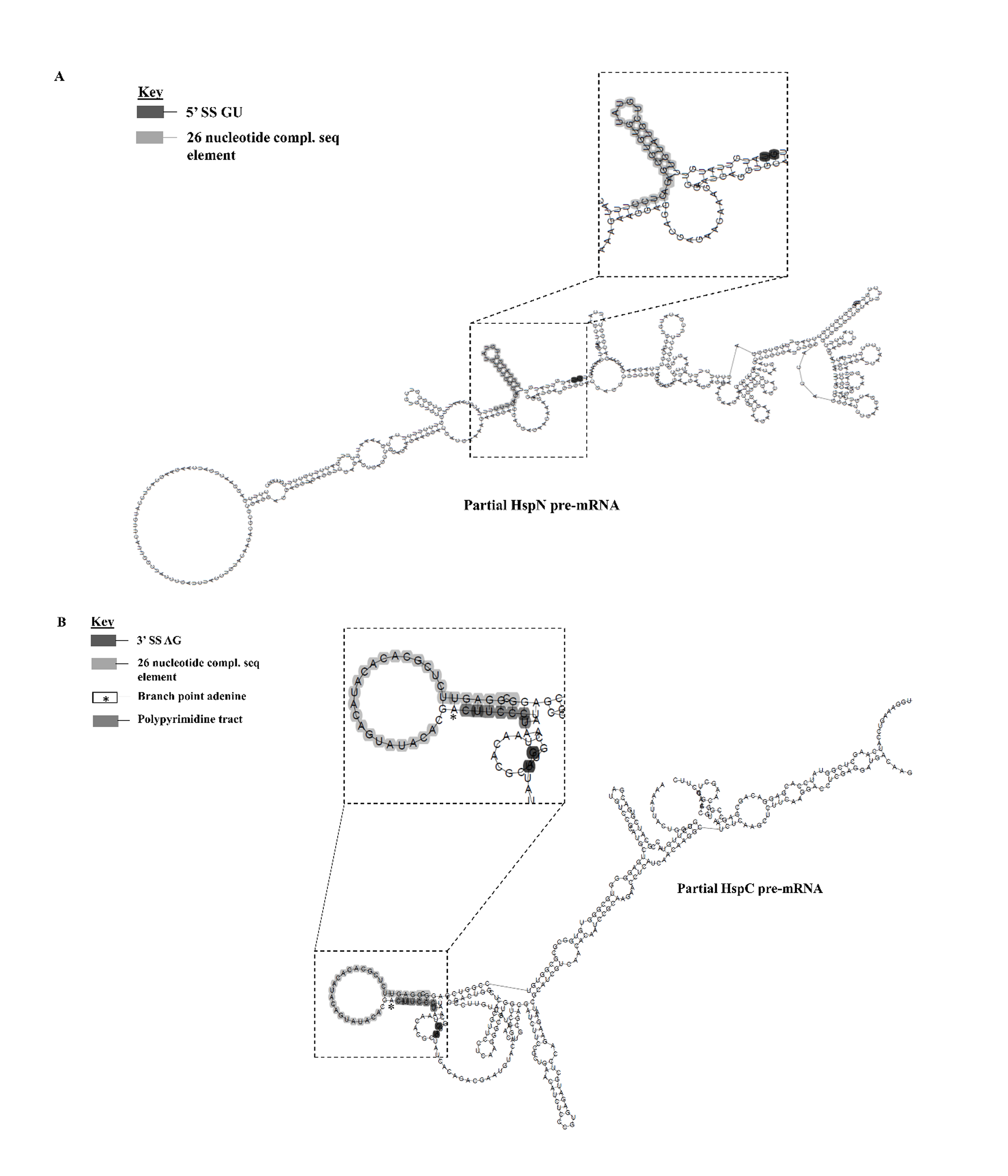

Supplement: S4 Fig — A) Stem loop enlarged in the inset harbors the 26 nt complementary functional sequence in partial HspN pre-mRNA. B) Stem loop enlarged in the inset harbors the 26 nt complementary sequence, branch point adenine and polypyrimidine tract functional sequences in partial HspC pre- mRNA. (TIF) [file pntd.0009810.s004.tif]

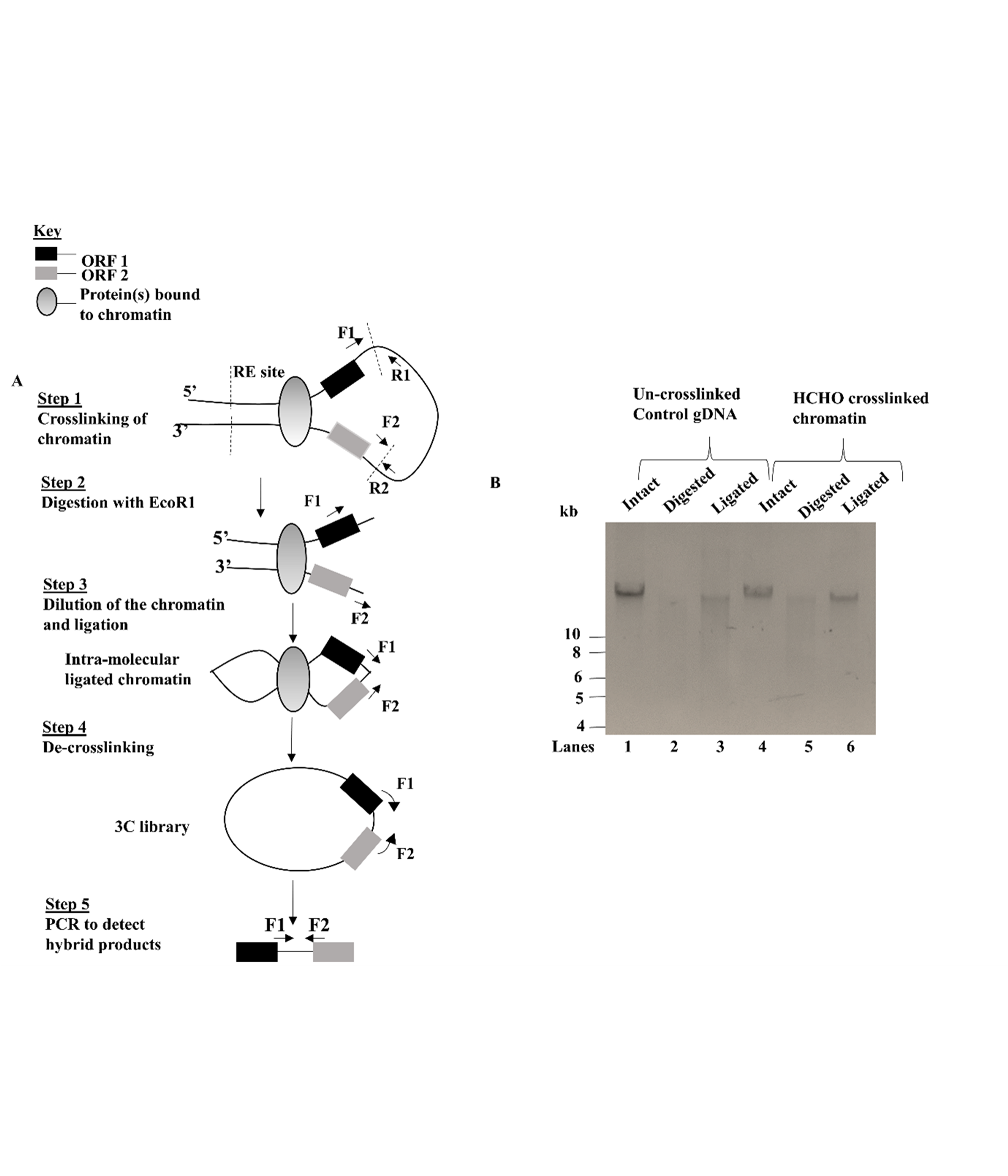

Supplement: S5 Fig — A) Schematic representation of 3C workflow. The chromatin conformation in the nuclei of 0.5 × 10^8^ log phase Giardia trophozoites were fixed with 1.5% formaldehyde. Same number of cells (0.5 × 10^8^) were not treated with formaldehyde and served as un-crosslinked control. Nuclei were isolated from both cross-linked and un-crosslinked cells. Chromatin was obtained from formaldehyde fixed cells by lysing the nuclear membrane. Crosslinked chromatin was then digestion with EcoR1. Digestion was followed by dilution (4 folds) to promote intramolecular ligation of cross-linked chromatin over intermolecular ligation to determine interacting loci in the fixed chromatin. Ligation was followed by reversal of crosslinks and identification of interacting loci on the chromatin using locus specific primers by semi-quantitative polymerase chain reaction. Genomic DNA was extracted from control un-crosslinked nuclei, digested with EcoR1 and ligated without dilution to promote random ligations. B) Agarose gel profile of intact, digested and ligated gDNA from un-crosslinked control chromatin and formaldehyde cross-linked chromatin. Lanes 1 and 4 show the intact control gDNA and formaldehyde fixed chromatin, lanes 2 and 5 show digested control gDNA and cross-linked chromatin; and lanes 3 and 6 show randomly ligated control gDNA without dilution and formaldehyde cross-linked chromatin post dilution. (TIF) [file pntd.0009810.s005.tif]

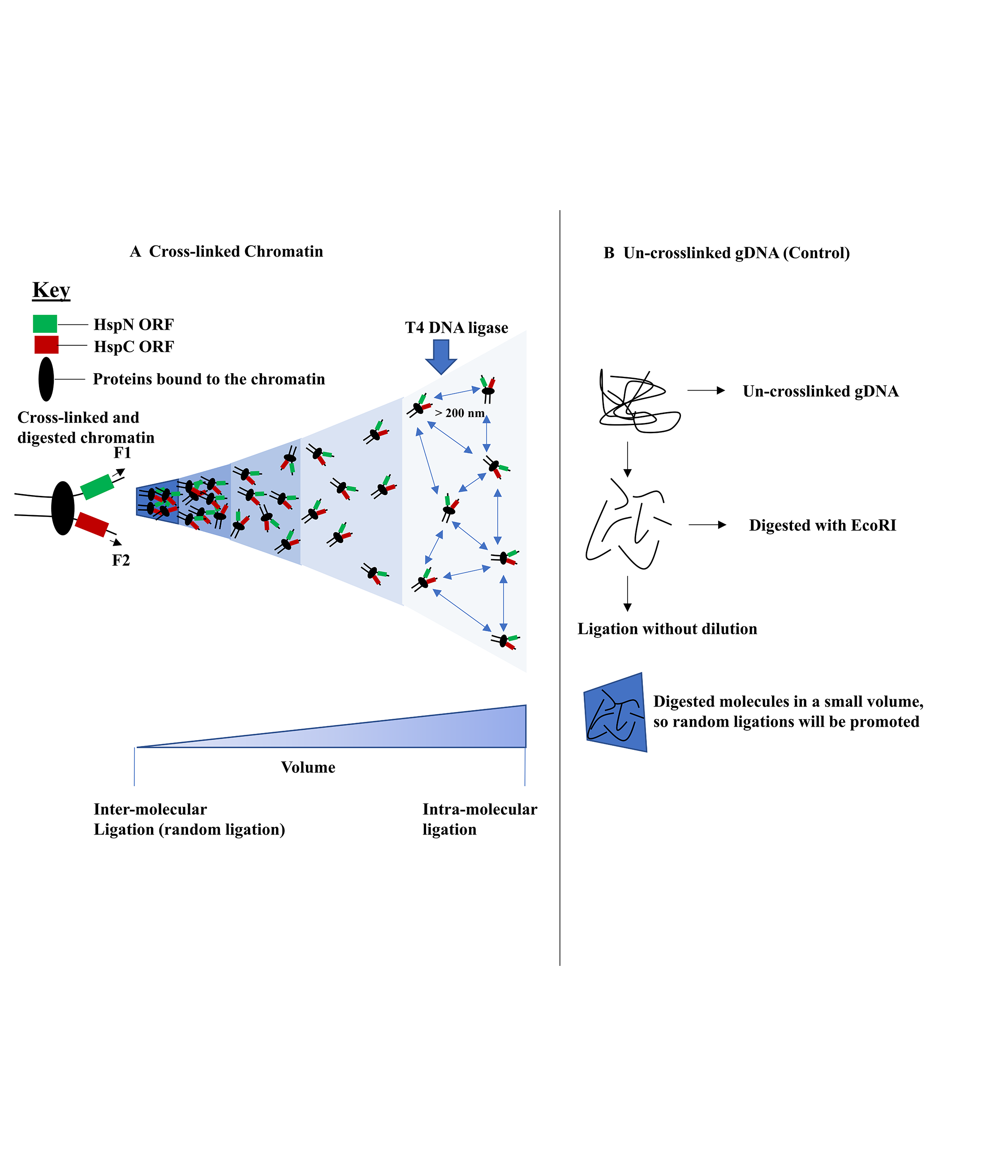

Supplement: S6 Fig — Black lines in the enlarged digested species represents chromatin. Green rectangle represents HspN ORF, red rectangle represents HspC ORF and black oval represents proteins bound to the chromatin. A) Formaldehyde cross-linked chromatin upon digestion is subjected to several fold dilution. Upon dilution, the digested molecules remain constant however, the volume would increase and therefore the intermolecular distance between the different digested species. When T4 DNA ligase is introduced at a particular dilution when the intermolecular distance increases beyond 200 nm, intramolecular ligation would be preferred over intermolecular ligation. 3C technology relies on detecting the hybrids that are enriched in the 3C library. B) Un-crosslinked extracted Giardia genomic DNA was utilized for negative control experiment. When genomic DNA was purified from the proteins, the DNA was not constrained in any structure. However, it was only contiguous stretch of nucleotides without proteins constraining it into any structure. The extracted genomic DNA was digested and ligated using T4 DNA ligase without dilution. This would result in every digested species having equal probability of ligating with any other digested species in close proximity. Therefore, ligation without dilution would promote random ligation between the digested species in a small volume as opposed to intramolecular ligation promoted in the case of the crosslinked chromatin. (TIF) [file pntd.0009810.s006.tif]

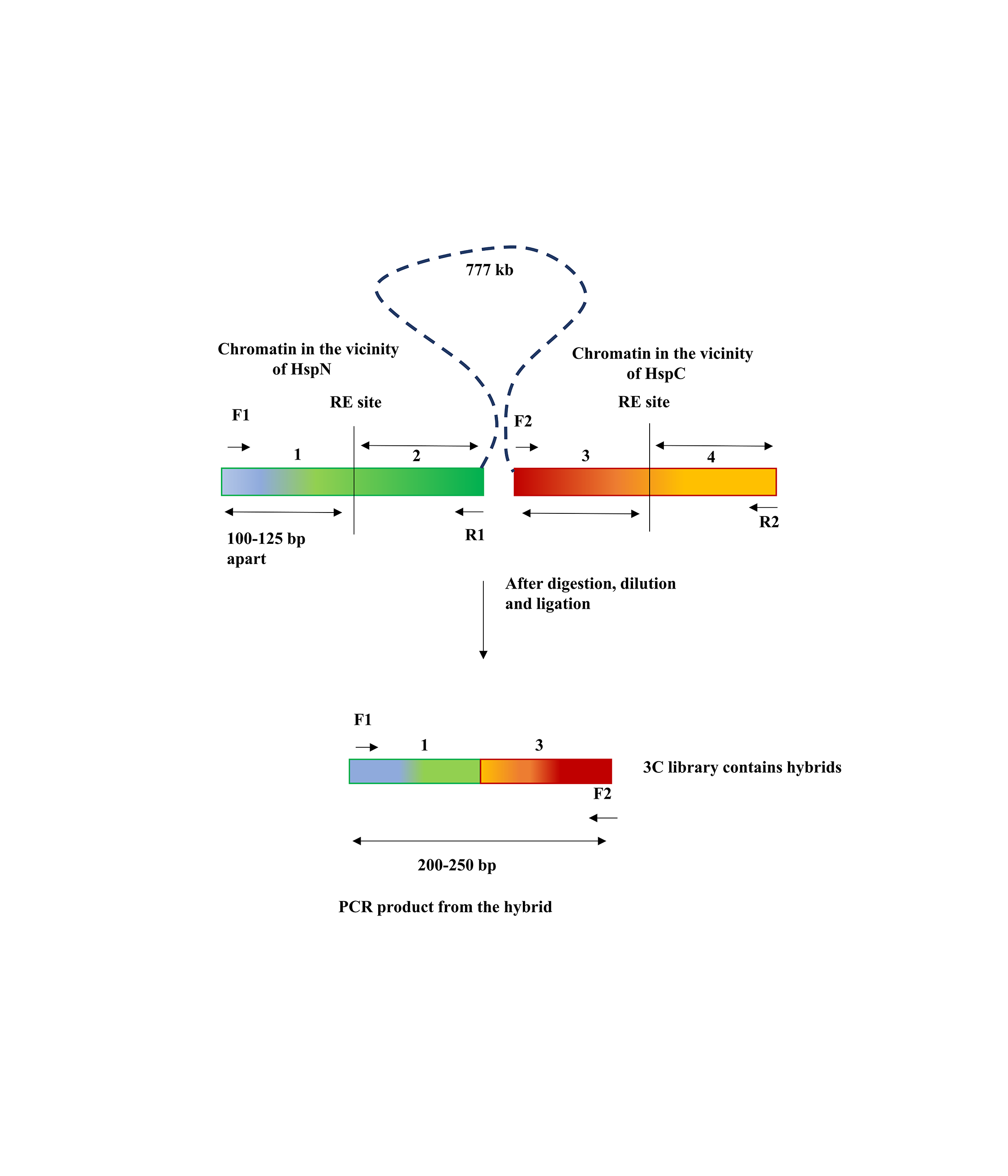

Supplement: S7 Fig — Schematic representation of primer design to detect different hybrids between loci. The blueish green rectangle represents chromatin in the vicinity of HspN and the yellowish red rectangle represents chromatin in the vicinity of HspC. The vertical dotted lines represent the EcoRI recognition sites close to HspN and HspC. For simplicity, only two EcoRI recognition sites/two EcoRI loci have been illustrated. However, to examine long range interaction between the two ORFs, 13 different loci (including the two recognition sites shown) were studied. Primers were designed flanking the EcoRI recognition sites. F1, R1 and F2, R2 represents flanking primers close to the EcoRI sites in the vicinity of HspN and HspC. Upon digestion, dilution and ligation of the chromatin different segments; 1, 2, 3 and 4 could ligate to each other in different combinations. Segment 1 could ligate to 3 and/or 4. Segment 2 could ligate to 3 and/or 4. To detect chromatin hybrids formed between segment 1 with segment 3 as shown, we used F1 and F2 as the primers and examined its enrichment using semi-quantitative PCR. (TIF) [file pntd.0009810.s007.tif]

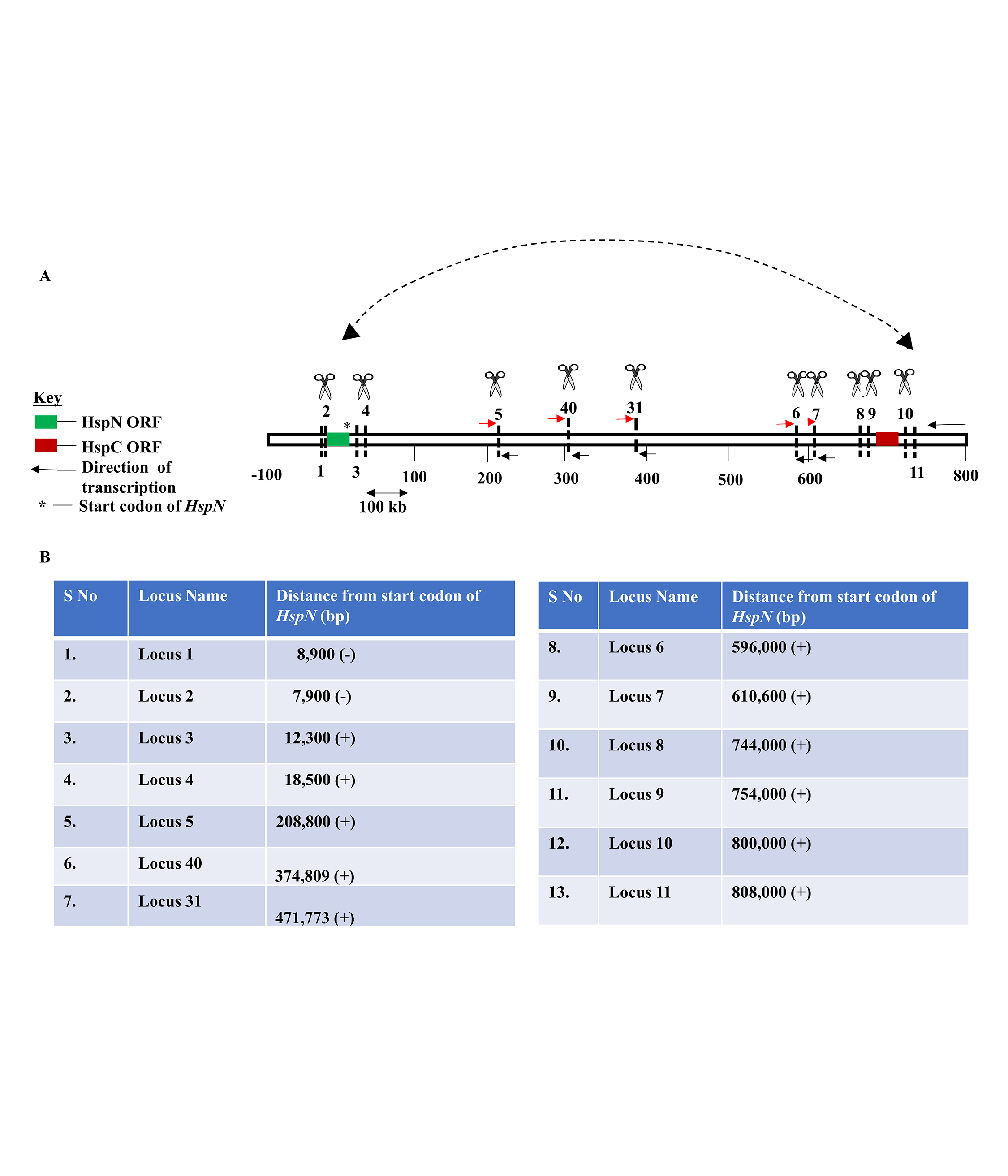

Supplement: S8 Fig — A) The different vertical lines represent the different EcoRI sites chosen for examining physical proximity of HspN and HspC on chromosome 5. The EcoRI sites are designated uniquely e.g., 1, 2, 3, 4, 5, 40, 31 etc., which are indicated above each vertical lines. The key on right top indicates the genes under investigation, the EcoRI loci chosen for study highlighted with the scissor symbol and indicates the start codon of HspN. B) The table displays the distances of each EcoRI loci chosen for the study from the start codon of HspN. The minus (-) symbol denotes that the distance in bp is towards the left of HspN and the plus (+) symbol denotes that the distance in bp is towards the right of HspN. (TIF) [file pntd.0009810.s008.tif]

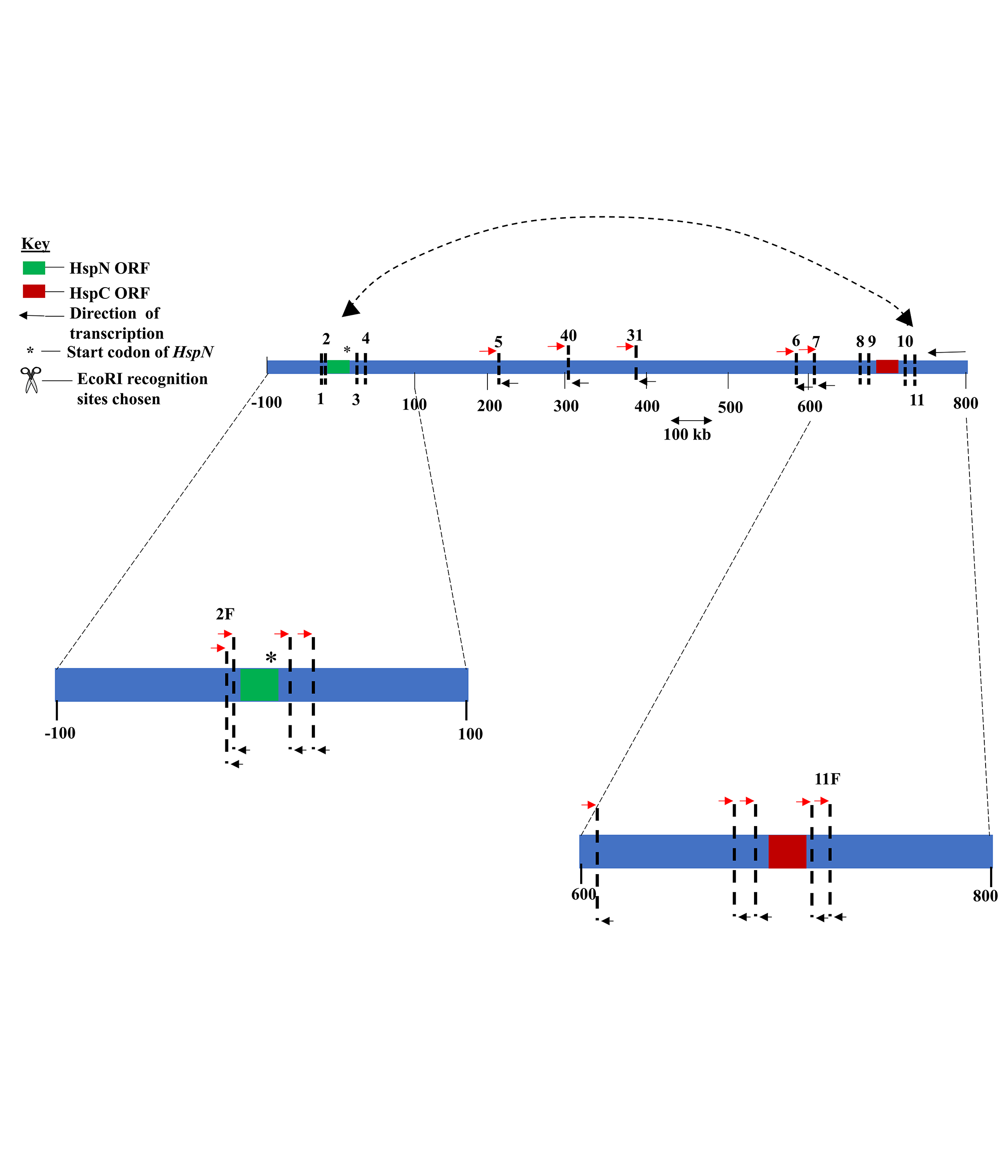

Supplement: S9 Fig — The numbers 1,2,3,4,5,40, 31, 6,7,8,9,10,11 represent the different EcoRI loci/EcoRI recognition sites which were investigated. The numbers below the map (-100, 200,300…) serves as a ruler and shows the distances of the different EcoRI loci from each other and from the genes under question. The red arrows represent the forward primers, and the black arrows represent the reverse primers. (TIF) [file pntd.0009810.s009.tif]

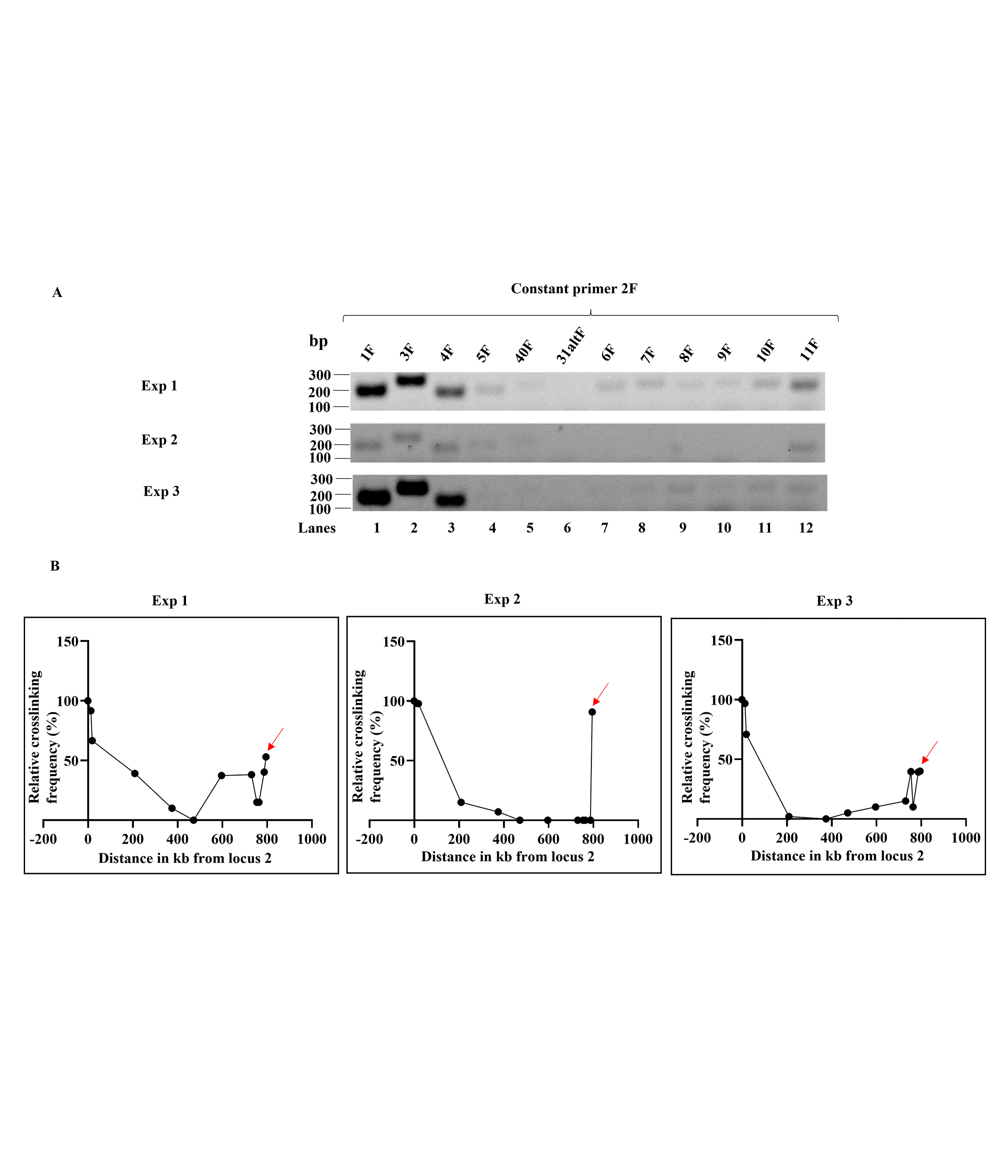

Supplement: S10 Fig — A) The three panels show the PCR products obtained with constant primer 2F with other forward primers designed across the different loci in vicinity and in between HspN and HspC ORFs chosen for this study. Loci 1 and 3 which are in proximity to locus 2 show intense bands of corresponding sizes. As the distance from locus 2 increases, the PCR amplicons fade out and the PCR signal then starts picking up from locus 6 gradually; increasing significantly at locus 11 confirming physical proximity and interaction between loci 2 and loci 11 which are proximal to HspN and HspC ORFs respectively. Similar trend of intensities was observed of the different hybrids in the 3C libraries from three different 3 experiments. C) The relative crosslinking frequency of locus 2 proximal to HspN was determined from the three experiments and plotted as a function of distance from the start codon of HspN. (TIF) [file pntd.0009810.s010.tif]

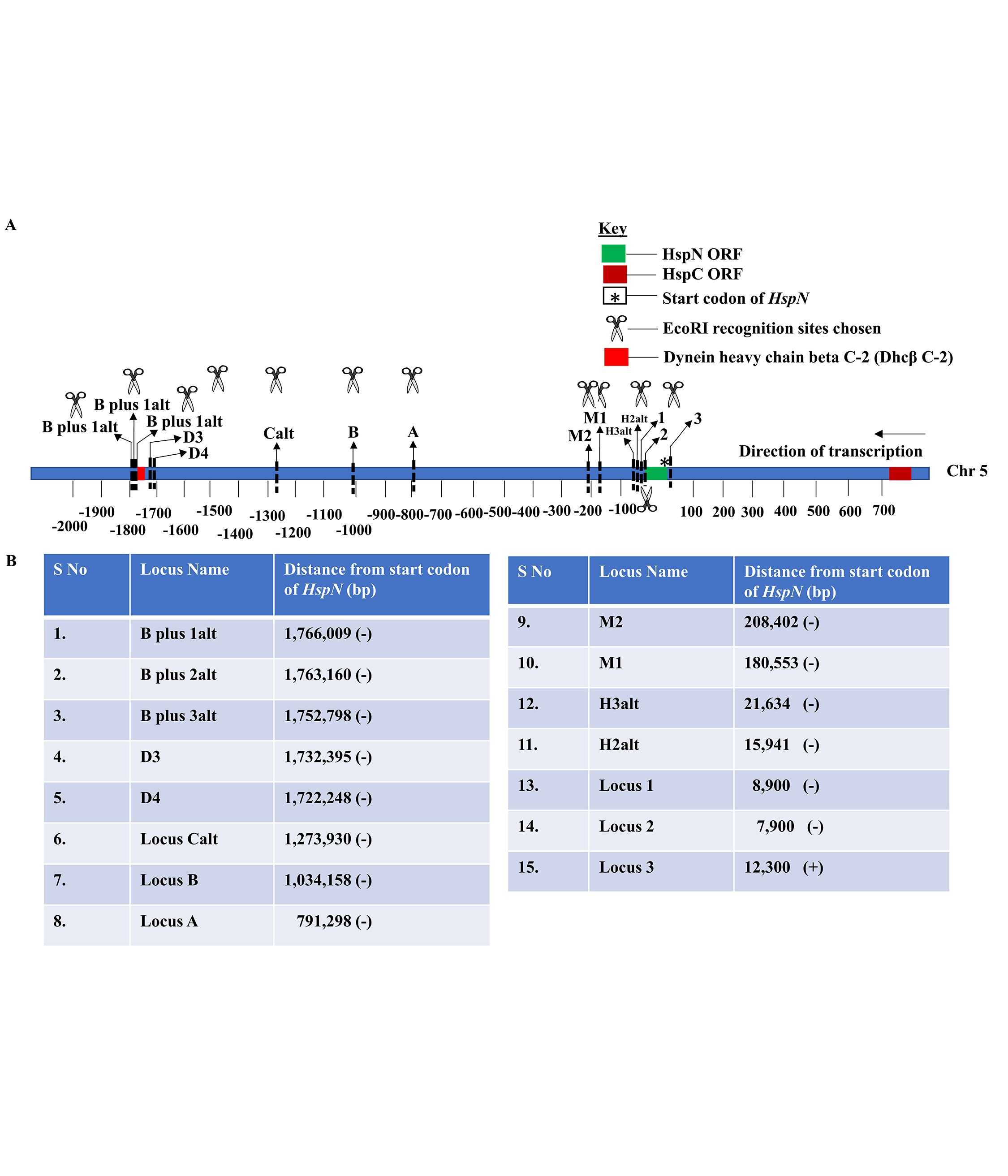

Supplement: S11 Fig — A) The different vertical lines represent the different EcoRI sites chosen for examining physical proximity of HspN and Dhcβ C-2 on chromosome 5. The EcoRI sites are designated uniquely e.g., B plus 1alt, B plus 2alt, B plus 3alt etc., which are indicated above each vertical line. The key on right top indicates the genes under investigation, the EcoRI loci chosen for study highlighted with the scissor symbol and the key also indicates the start codon of HspN. The numbers below the map (-2000, -1900, -1800, -1700 and so on) serves as a ruler and shows the distances of the different EcoRI loci from each other and from the genes under question. B) The table displays the distances of each EcoRI loci chosen for the study from the start codon of HspN. The minus (-) symbol denotes that the distance in bp is towards the left of HspN and the plus (+) symbol denotes that the distance in bp is towards the right of HspN. (TIF) [file pntd.0009810.s011.tif]

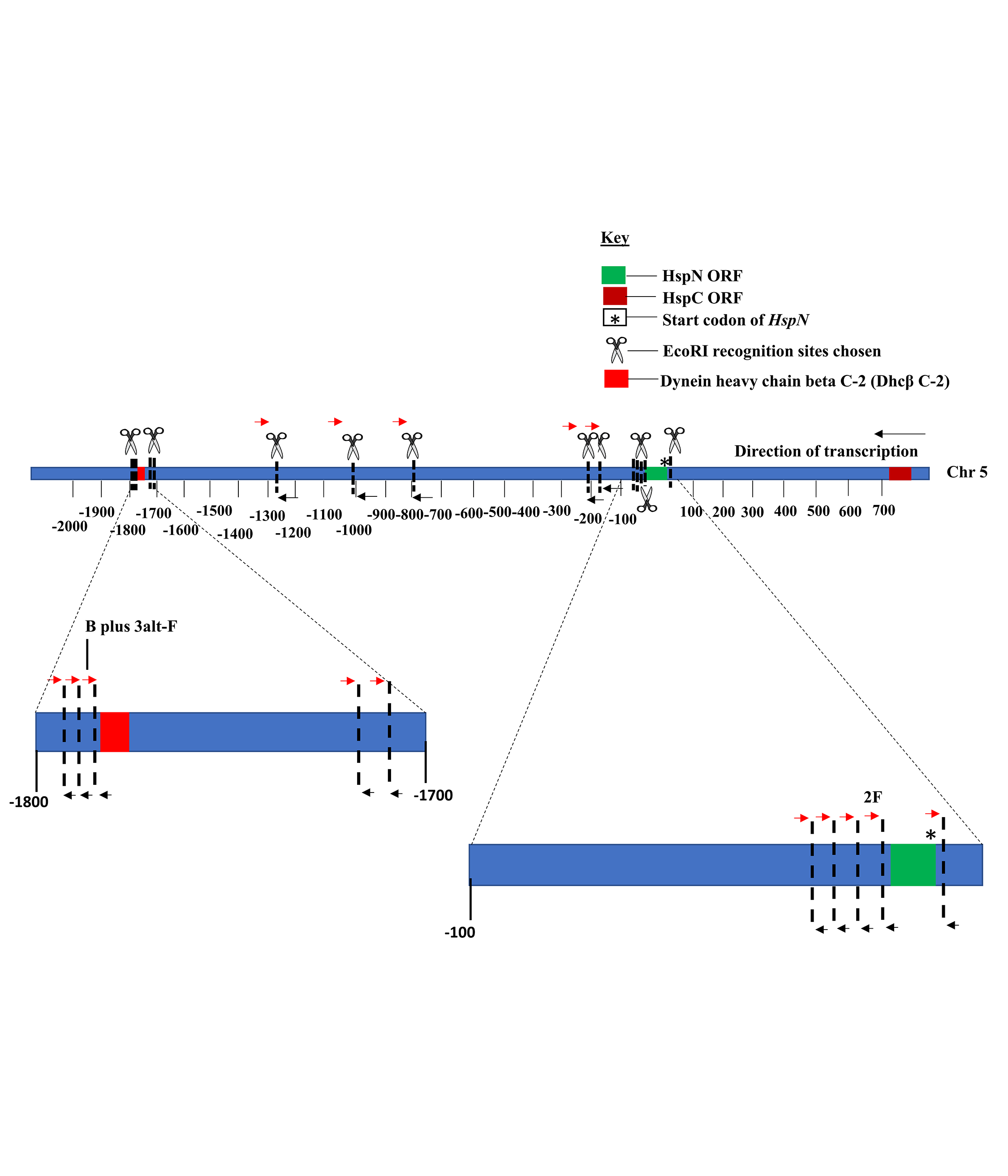

Supplement: S12 Fig — The red arrows represent the forward primer and the black arrows represent the reverse primers. Please refer to S11 Fig to correlate EcoRI recognition sites with their corresponding names. (TIF) [file pntd.0009810.s012.tif]

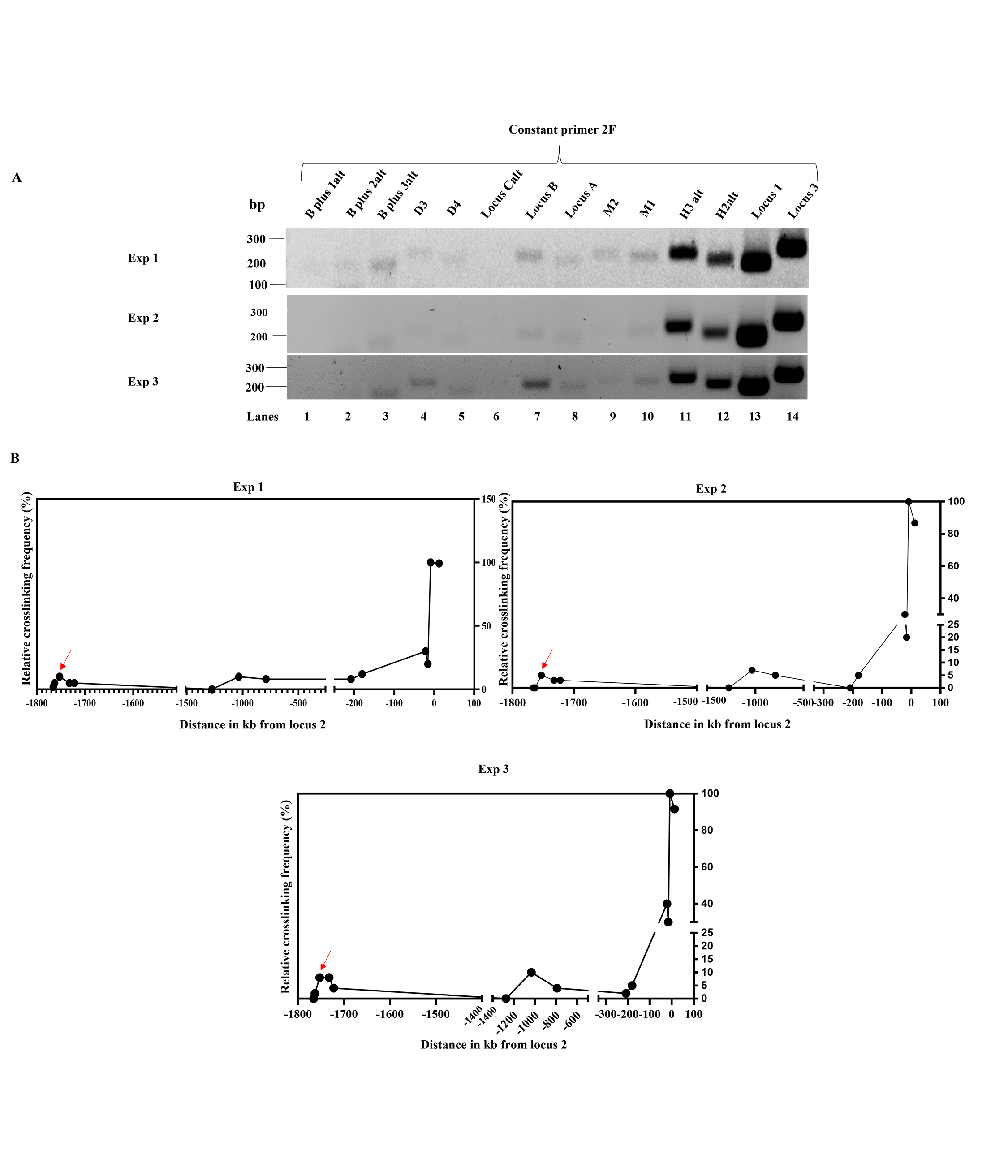

Supplement: S13 Fig — A) The panels show the PCR products obtained with constant primer 2F with other forward primers across the different loci in vicinity and in between HspN and Dhcβ C-2 ORFs. Loci 1 and 3 which are in proximity to locus 2 show intense bands of corresponding sizes. As the distance from locus 2 towards the Dhcβ C-2 increases, the PCR amplicons fade out and the PCR signal then picks up at locus B and then at locus B plus 3alt confirming physical proximity and interaction between loci 2 and B plus 3alt which are proximal to HspN and Dhcβ C-2 ORFs respectively. Similar trend of intensities was observed of the different hybrids in the 3C libraries from three different 3 experiments. C) The relative crosslinking frequency of locus 2 and locus B plus 3alt proximal to HspN and Dhcβ C-2 ORFs was determined from the three experiments and plotted as a function of distance from locus 2. (TIF) [file pntd.0009810.s013.tif]

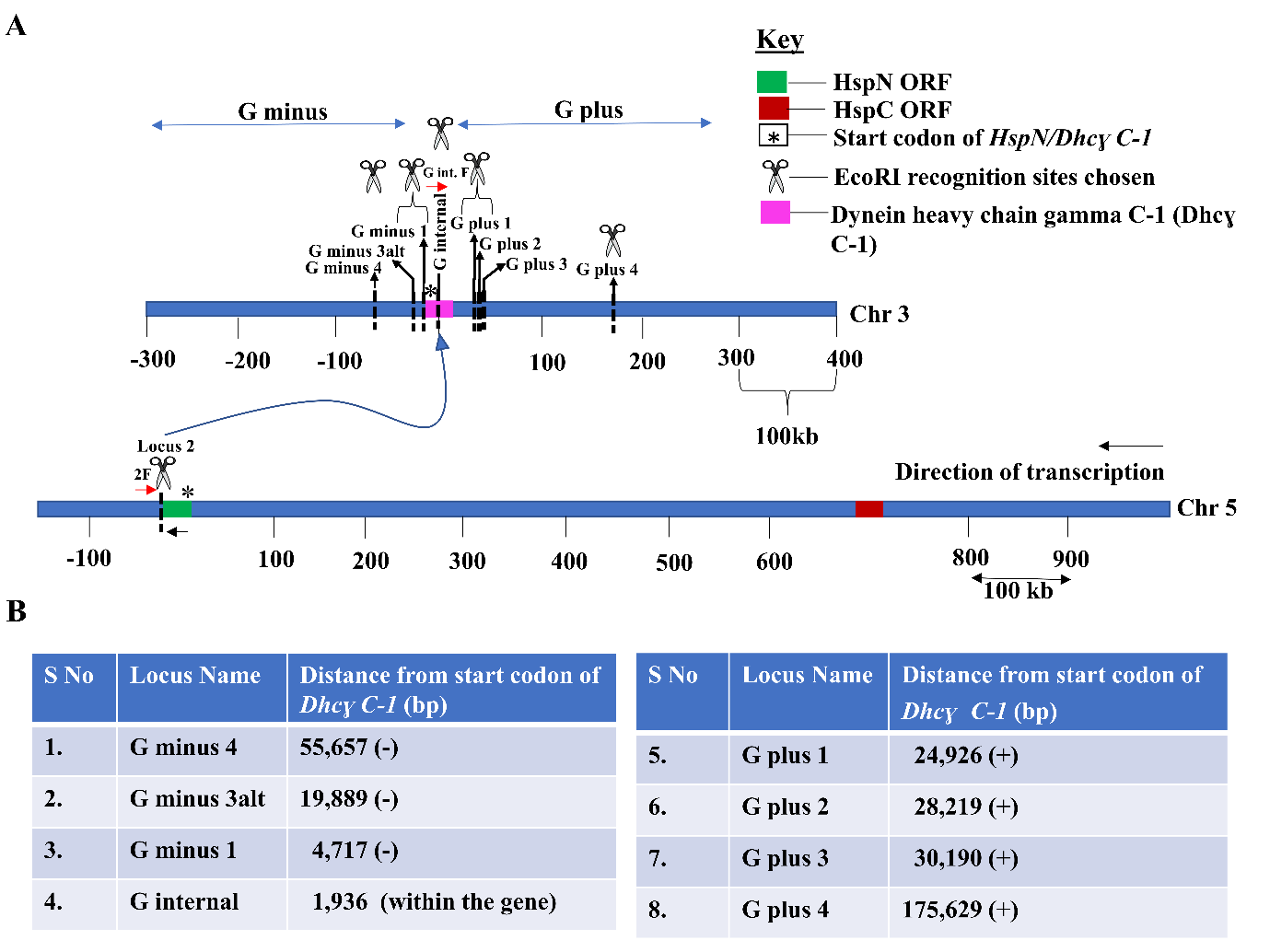

Supplement: S14 Fig — A) The different vertical lines represent the different EcoRI sites chosen for examining physical proximity of HspN on chromosome 5 and Dhcγ C-1 on chromosome 3. The EcoRI sites are designated uniquely e.g., G minus 4, G minus 3alt, G minus 1 etc., which are indicated above each vertical line. The key on right top indicates the genes under investigation, the EcoRI loci chosen for study highlighted with the scissor symbol and the key also indicates the start codon of HspN and Dhcɣ C-1. The numbers below the map of chromosome 3 (-300, -200, -100 and so on till 400) serve as ruler to show the relative genomic distances between the EcoRI loci on chromosome 3. B) The table displays the distances of each EcoRI loci chosen for the study from the start codon of Dhcɣ C-1. The minus (-) symbol denotes that the distance in bp is towards the left of Dhcɣ C-1 gene and the plus (+) symbol denotes that the distance in bp is towards the right of Dhcɣ C-1 gene. (DOCX) [file pntd.0009810.s014.docx]

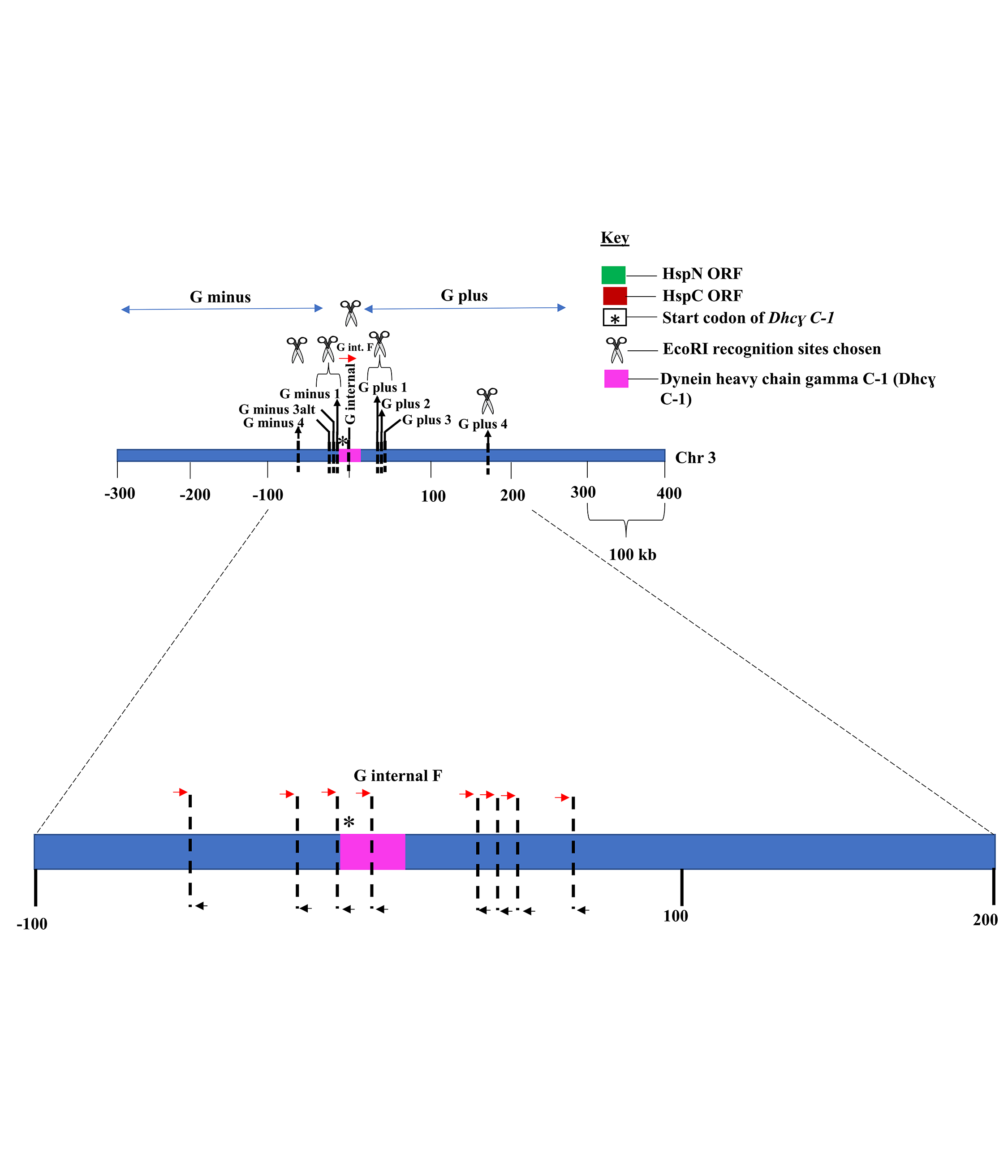

Supplement: S15 Fig — Schematic representation displaying the forward and reverse primers flanking each EcoRI site chosen to examine physical proximity between HspN on chromosome 5 and Dhcɣ C-1 on chromosome 3. The Figure shows the EcoRI recognition sites on chromosome 3. The red arrows represent the forward primers, and the black arrows represent the reverse primers. (TIF) [file pntd.0009810.s015.tif]

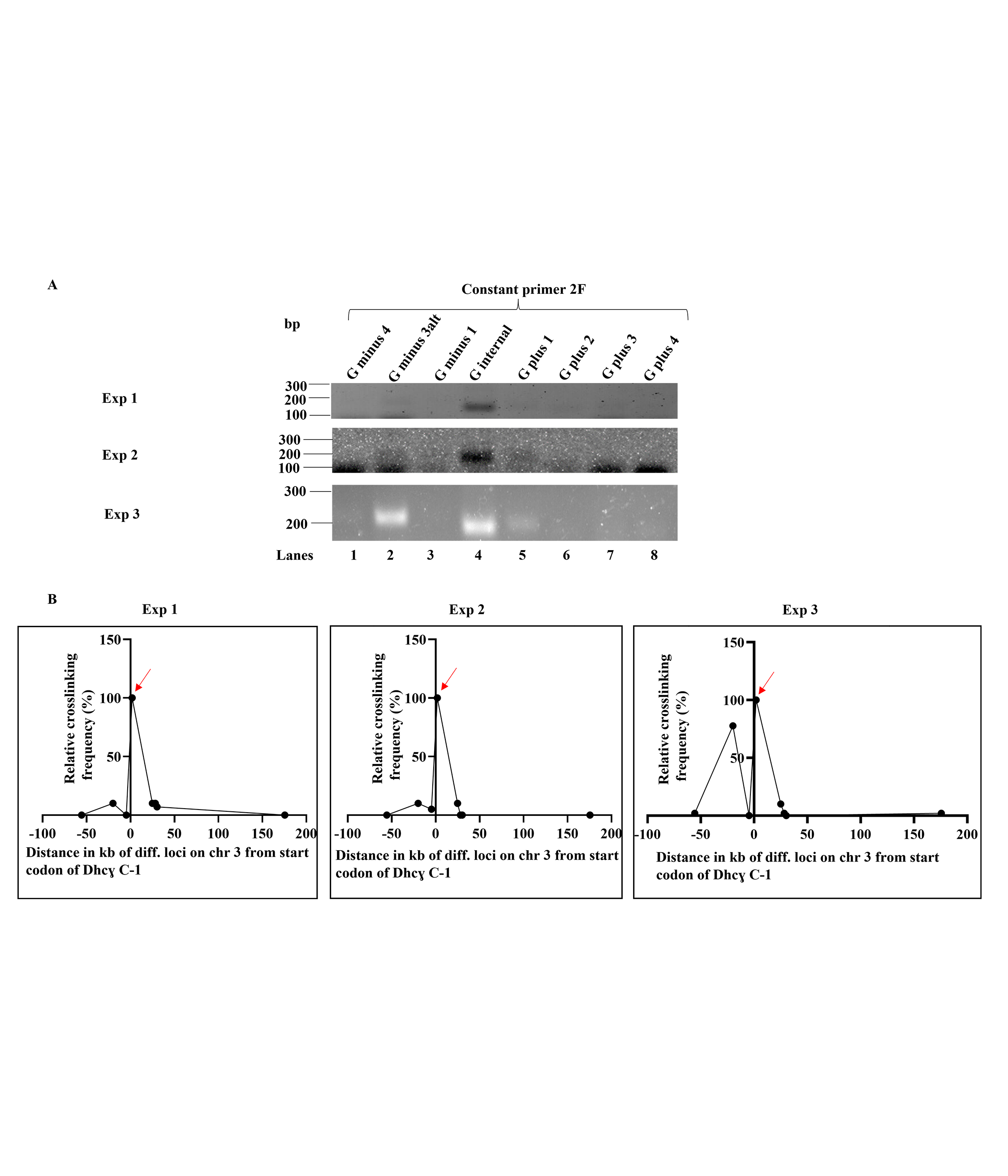

Supplement: S16 Fig — A) The panels show the PCR products obtained with constant primer 2F with other forward primers across the different loci in vicinity and within Dhcɣ C-1 ORF on chromosome 3 chosen for this study. Loci within Dhcɣ C-1 forms enriched hybrid 3C product with locus 2F in the 3C library preparation. Other loci on either side of Dhcɣ C-1 show very faint or no amplification with 2F confirming interaction of HspN with Dhcɣ C-1 on chromosome 3. Similar trend of intensities was observed of the different hybrids in the 3C libraries from three different 3 experiments. B) The relative crosslinking frequency of locus 2 and locus internal to Dhcɣ C-1 (G internal) along with the loci chosen upstream and downstream of Dhcɣ C-1 was determined from the three experiments and plotted as a function of distance from the start codon of Dhcɣ C-1. (TIF) [file pntd.0009810.s016.tif]

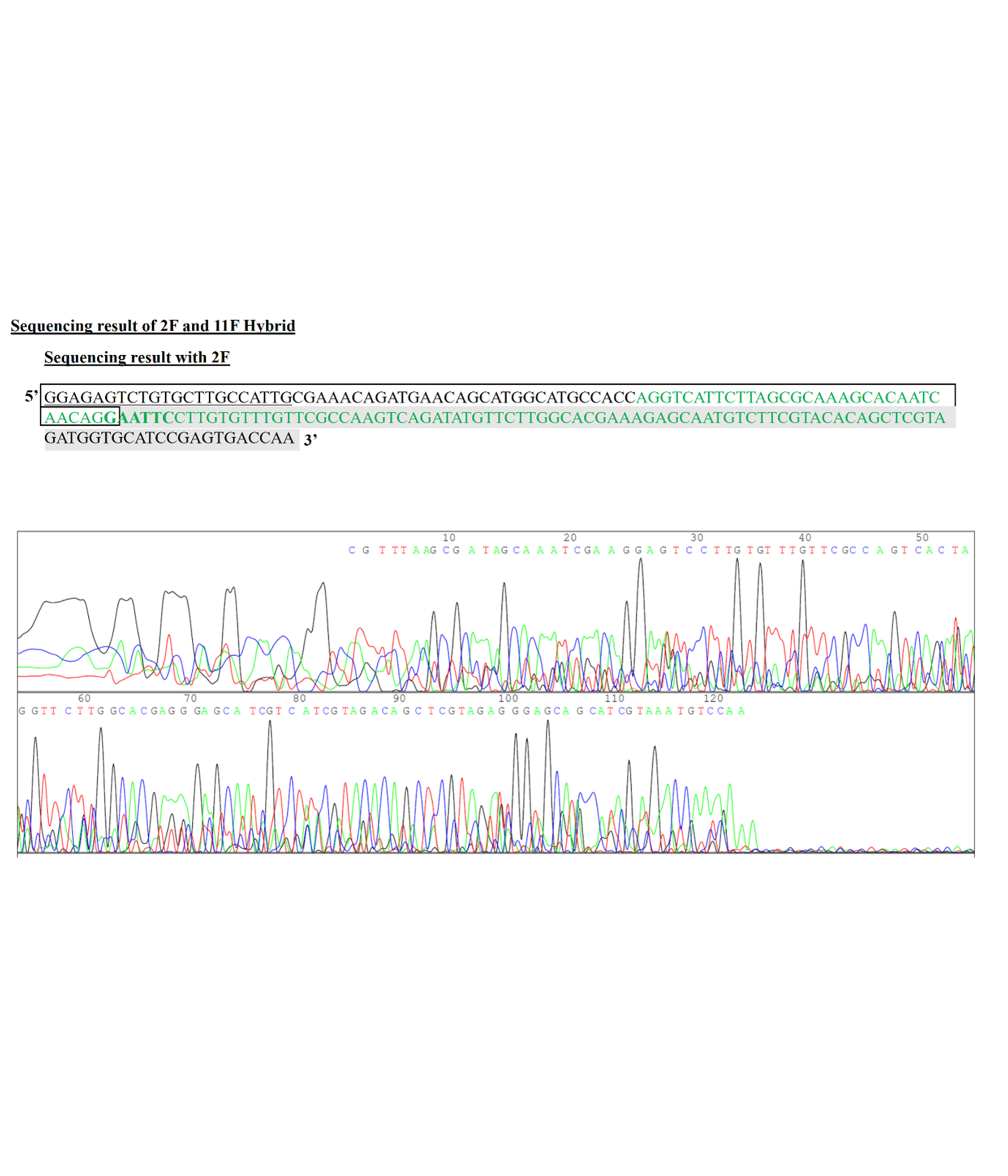

Supplement: S17 Fig — The enriched hybrid formed from locus 2 and locus 11 (Fig 2B, panel 1-lane 12) was excised and subjected to Sanger’s DNA sequencing with 2F primer. Sequencing result with 2F as the primer could detect the presence of sequences from locus 2 and locus 11 which confirms the presence of this hybrid in the enriched band. (TIF) [file pntd.0009810.s017.tif]

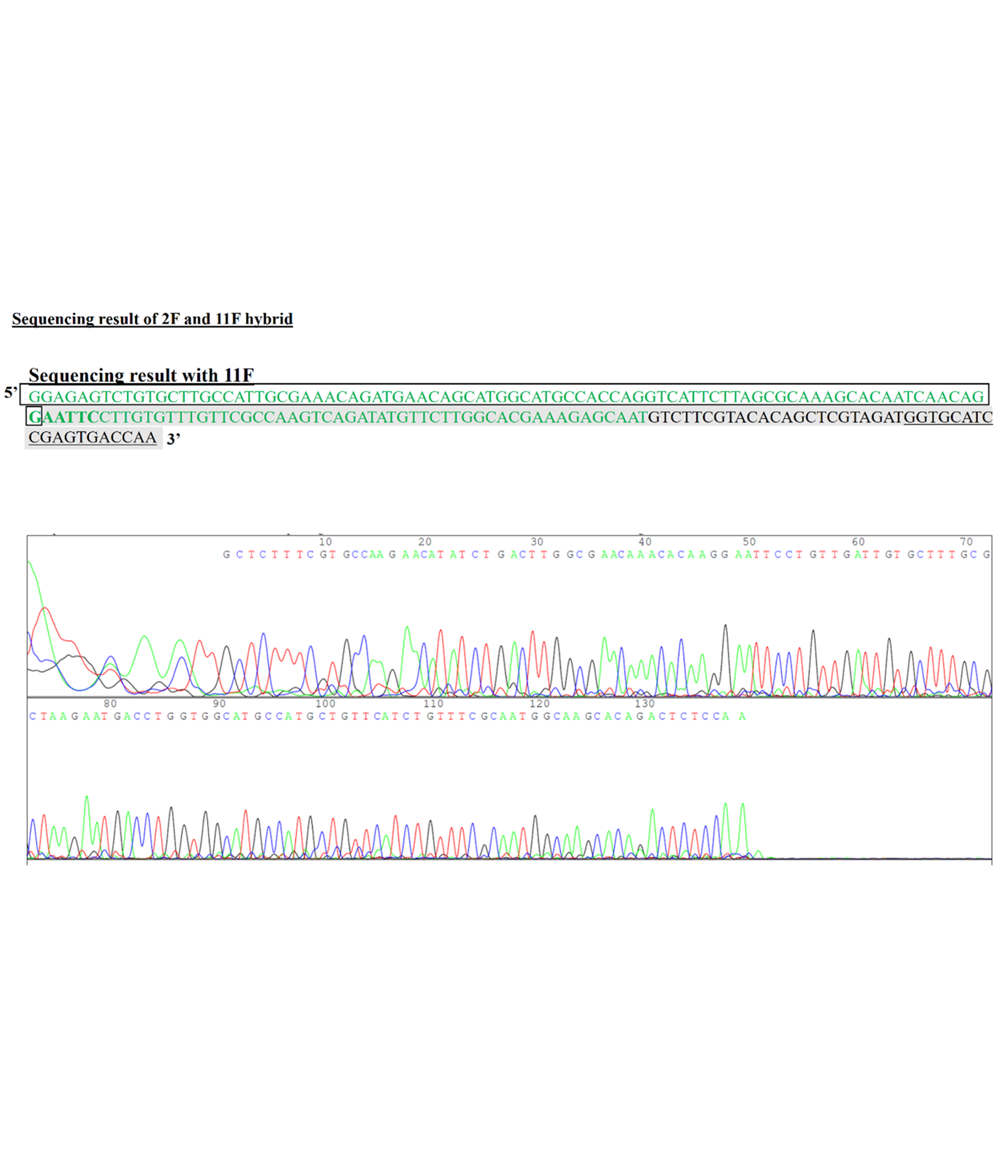

Supplement: S18 Fig — The enriched hybrid formed from locus 2 and locus 11 (Fig 2B, panel 1-lane 12) was excised and subjected to Sanger’s DNA sequencing with 11F primer. Sequencing result with 11F as the primer could detect the presence of sequences from locus 11 and locus 2 which confirms the presence of the hybrid in the enriched band. (TIF) [file pntd.0009810.s018.tif]

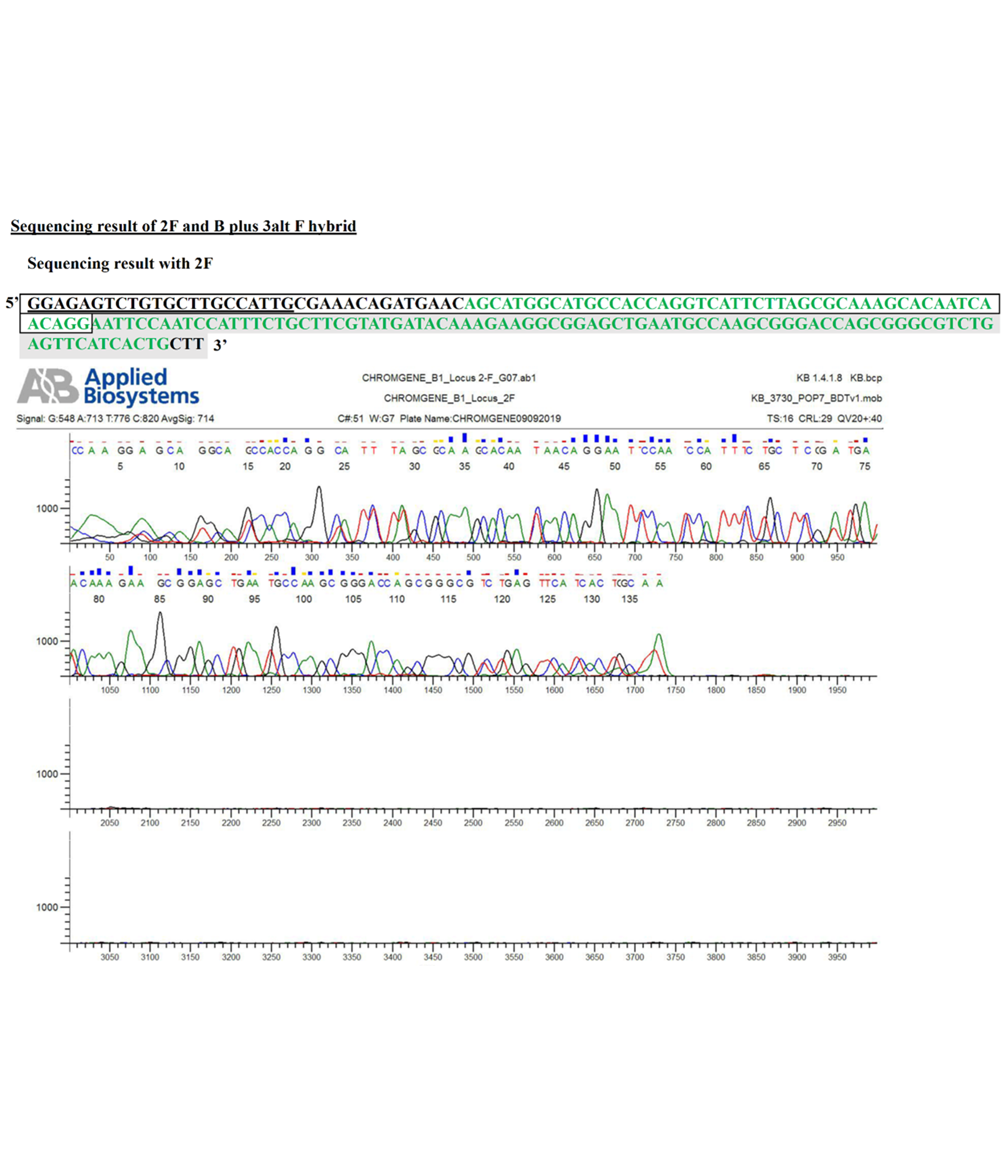

Supplement: S19 Fig — The enriched hybrid formed from locus 2 and locus B plus 3alt (Fig 4B, panel 1-lane 3) was excised and subjected to sanger’s DNA sequencing with 2F primer. Sequencing result with 2F as the primer could detect the presence of sequences from locus 2 and locus B plus 3alt which confirms the presence of hybrid. (TIF) [file pntd.0009810.s019.tif]

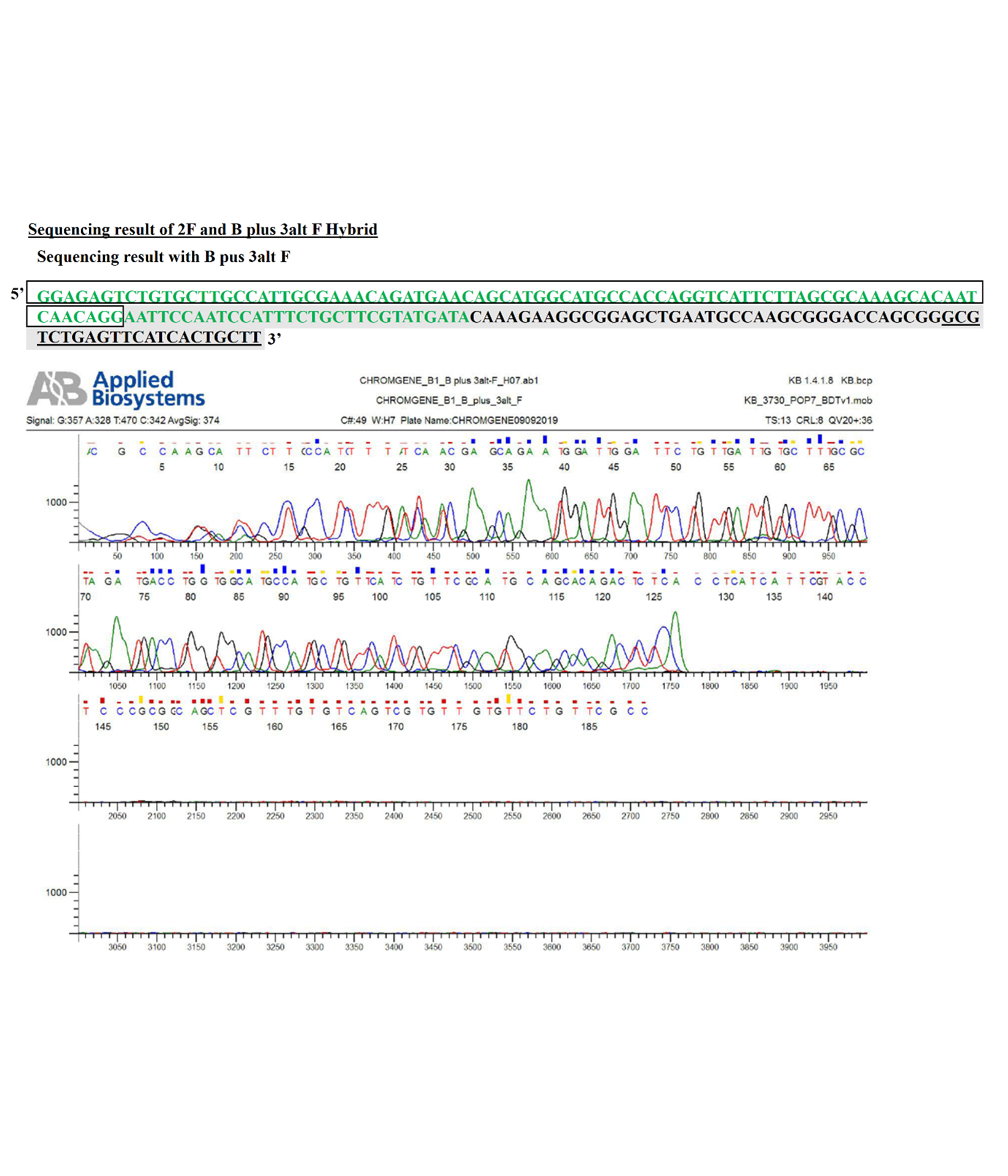

Supplement: S20 Fig — The enriched hybrid formed from locus 2 and locus B plus 3alt (Fig 4B, panel 1-lane 3) was excised and subjected to sanger’s DNA sequencing with B plus 3alt-F primer. Sequencing result with B plus 3alt-F as the primer could detect the presence of sequences from locus B plus 3alt and locus 2 which confirms the presence of hybrid. (TIF) [file pntd.0009810.s020.tif]

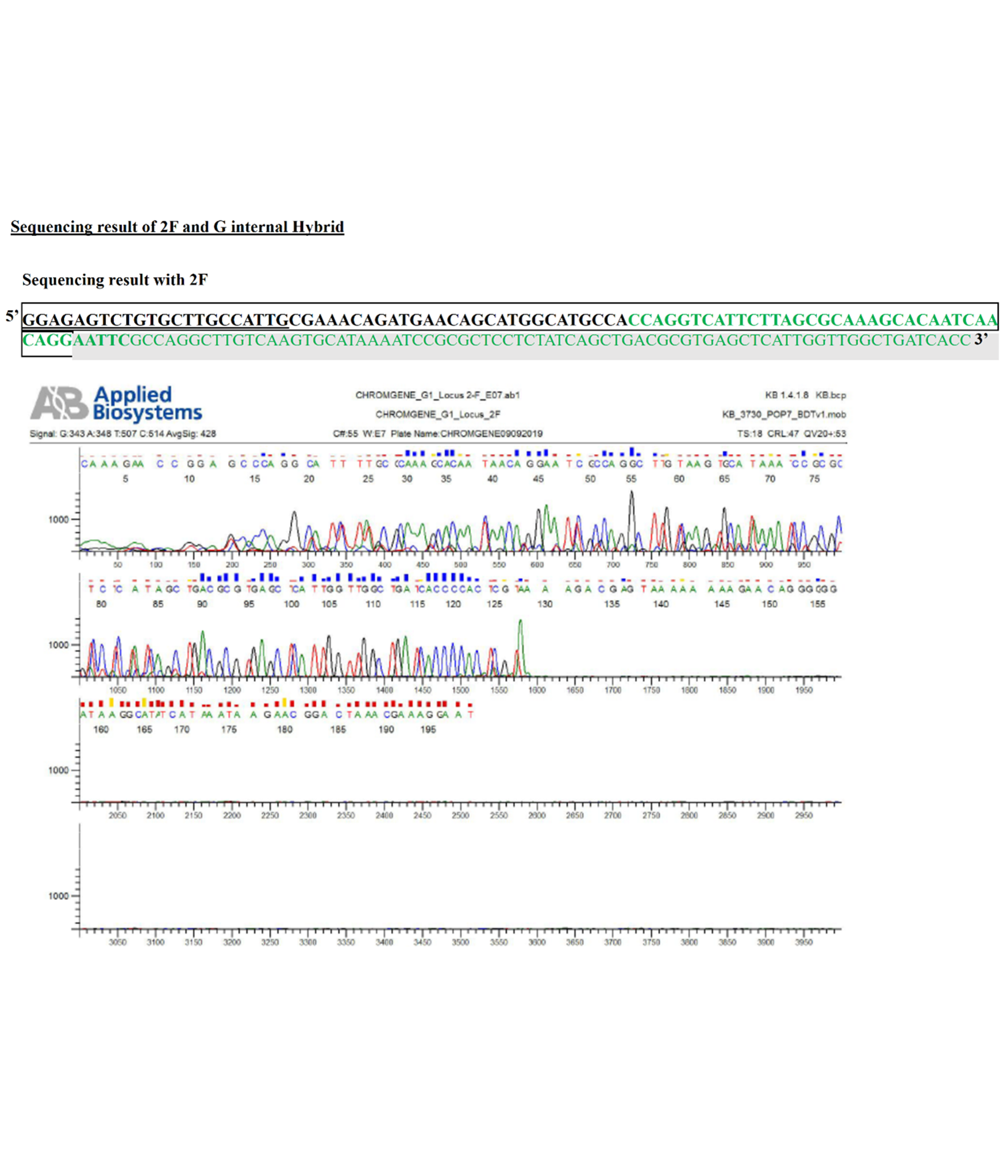

Supplement: S21 Fig — The enriched hybrid formed from locus 2 and locus G internal (Fig 4E, panel 1 lane 4) was excised and subjected to sanger’s DNA sequencing with 2F primer. Sequencing result with 2F as the primer could detect the presence of sequences from locus 2 and locus G internal which confirms the presence of hybrid. (TIF) [file pntd.0009810.s021.tif]

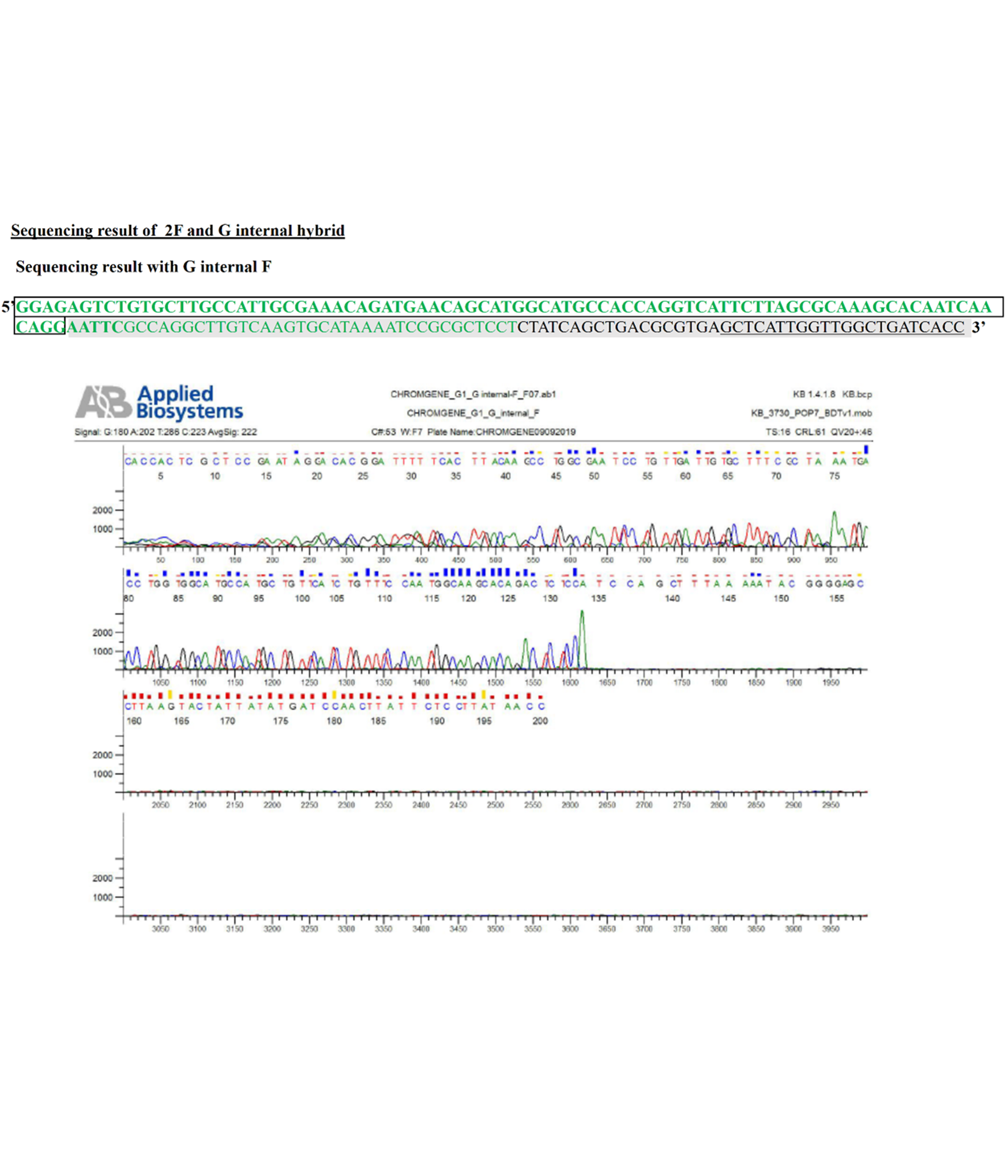

Supplement: S22 Fig — The enriched hybrid formed from locus 2 and locus G internal (Fig 4E, panel 1 lane 4) was excised and subjected to sanger’s DNA sequencing with G internal-F primer. Sequencing result with G internal-F as the primer could detect the presence of sequences from locus G internal and locus 2 which confirms the presence of hybrid. (TIF) [file pntd.0009810.s022.tif]

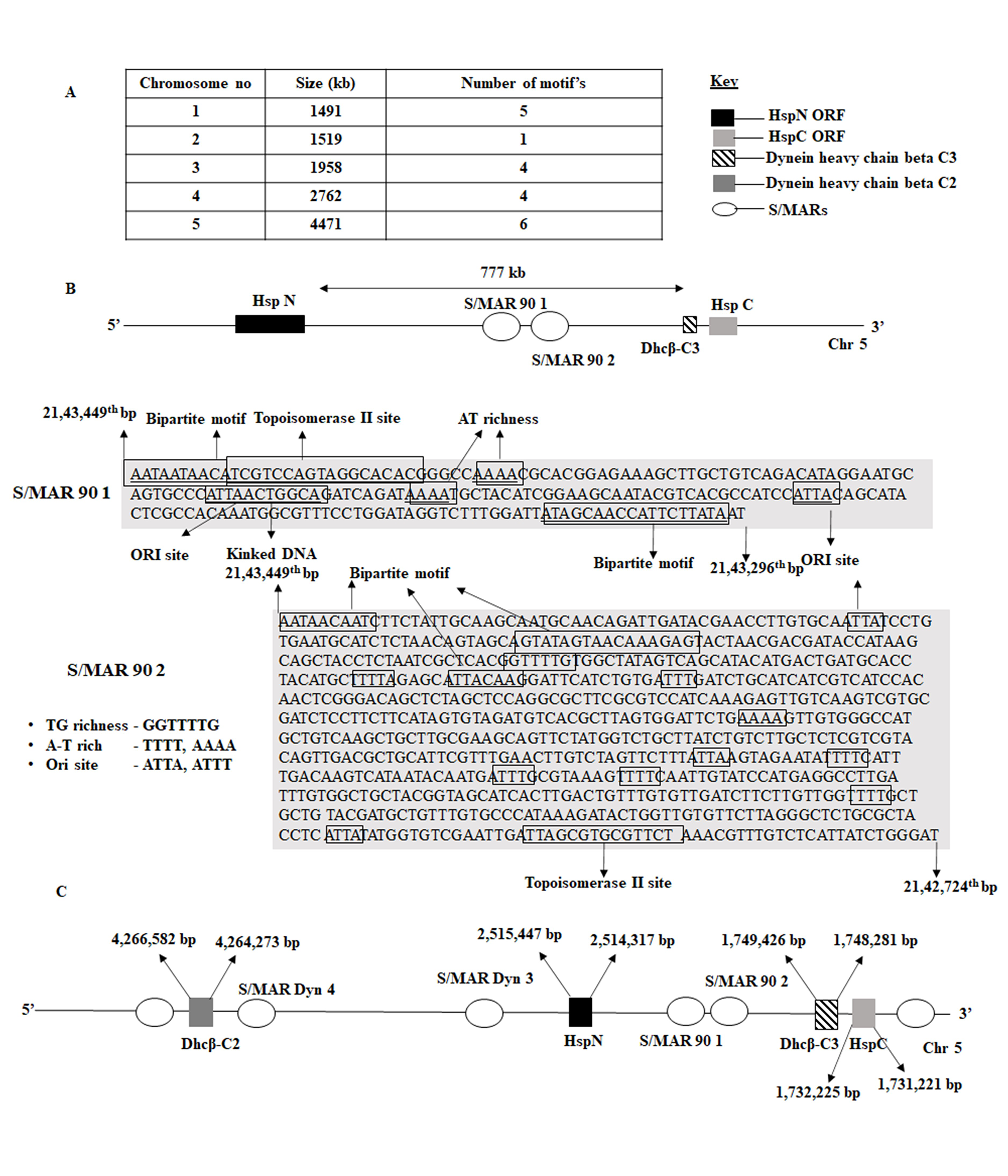

Supplement: S23 Fig — A) S/MAR like DNA sequence elements predicted by marscan across all the chromosomes of Giardia lamblia assemblage A WB C6. G. lamblia genomic DNA was investigated for the presence of S/MAR rules reported by Singh et al., 2000. Marscan online tool as well as in-house script predicted 20 S/MAR like DNA elements across all the 5 chromosomes of Giardia. B) Marscan predicts the presence of potential S/MAR like element in between HspN and HspC ORFs. Simplified schematic displays the presence of all the S/MAR rules i) bipartite motif, ii) general AT richness, iii) ORI site, iv) topoisomerase binding site, v) kinked DNA followed by a 204 bp and 725 bp region between HspN and HspC ORFs on chromosome 5 [32]. C) Schematic representation of the distribution of S/MARs predicted bioinformatically on Chromosome 5 in between the trans-spliced genes. (TIF) [file pntd.0009810.s023.tif]

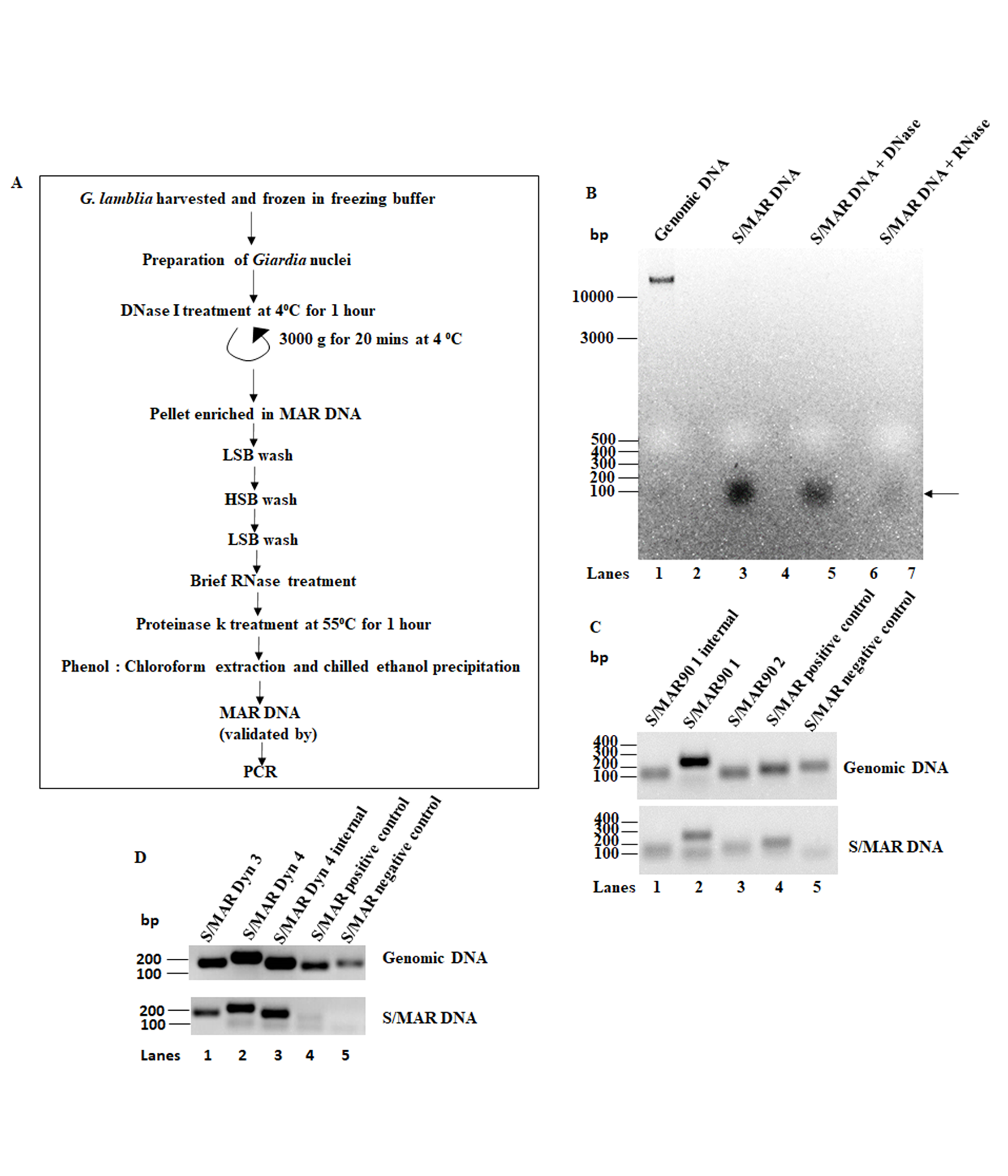

Supplement: S24 Fig — A) Schematic representation of the workflow for enrichment of S/MAR like DNA elements from G. lamblia. B) Size distribution and identity of enriched S/MAR DNA on 1.2% agarose gel. Lane 1 shows intact genomic DNA from Giardia lamblia; lane 3 shows enriched S/MAR DNA ranging in size upto approximately 200 bps. Lane 5 shows enriched S/MAR DNA elements post DNAse treatment; lane 7 shows S/MAR DNA element post RNase treatment. C) PCR validates the presence of bioinformatically predicted S/MAR elements between HspN and HspC ORFs. Top panel shows PCR products obtained with the genomic DNA as the template. Lower panel displays PCR validation of predicted S/MARs. Lane 1 shows amplicon of 106 bps with internal primers for the predicted 204 bp S/MAR element, lane 2 shows amplicon of 204 bps with primers designed at the ends of the predicted S/MAR sequence, lane 3 shows the PCR product of another predicted S/MAR of 108 bps, Lane 4 shows presence of previously reported S/MAR by Padmaja et al [19]; thus serves as the positive control. Lane 5 serves as the negative control. D) PCR validates the presence of bioinformatically predicted S/MAR elements between HspN and Dhcβ-C2. Top panel shows PCR products obtained with the genomic DNA as the template. Lower panel displays PCR validation of predicted S/MARs. Lane 1 shows amplicon of 163 bps with primers for the predicted S/MAR element between HspN and Dhcβ C-2, lane 2 shows amplicon of 202 bps with primers designed for another predicted S/MAR sequence, lane 3 shows the PCR product obtained with internal primers for S/MAR Dyn 4 between HspN and Dhcβ C-2, Lane 4 shows presence of previously reported S/MAR, thus serves as the positive control. Lane 5 serves as the negative control. (TIF) [file pntd.0009810.s024.tif]

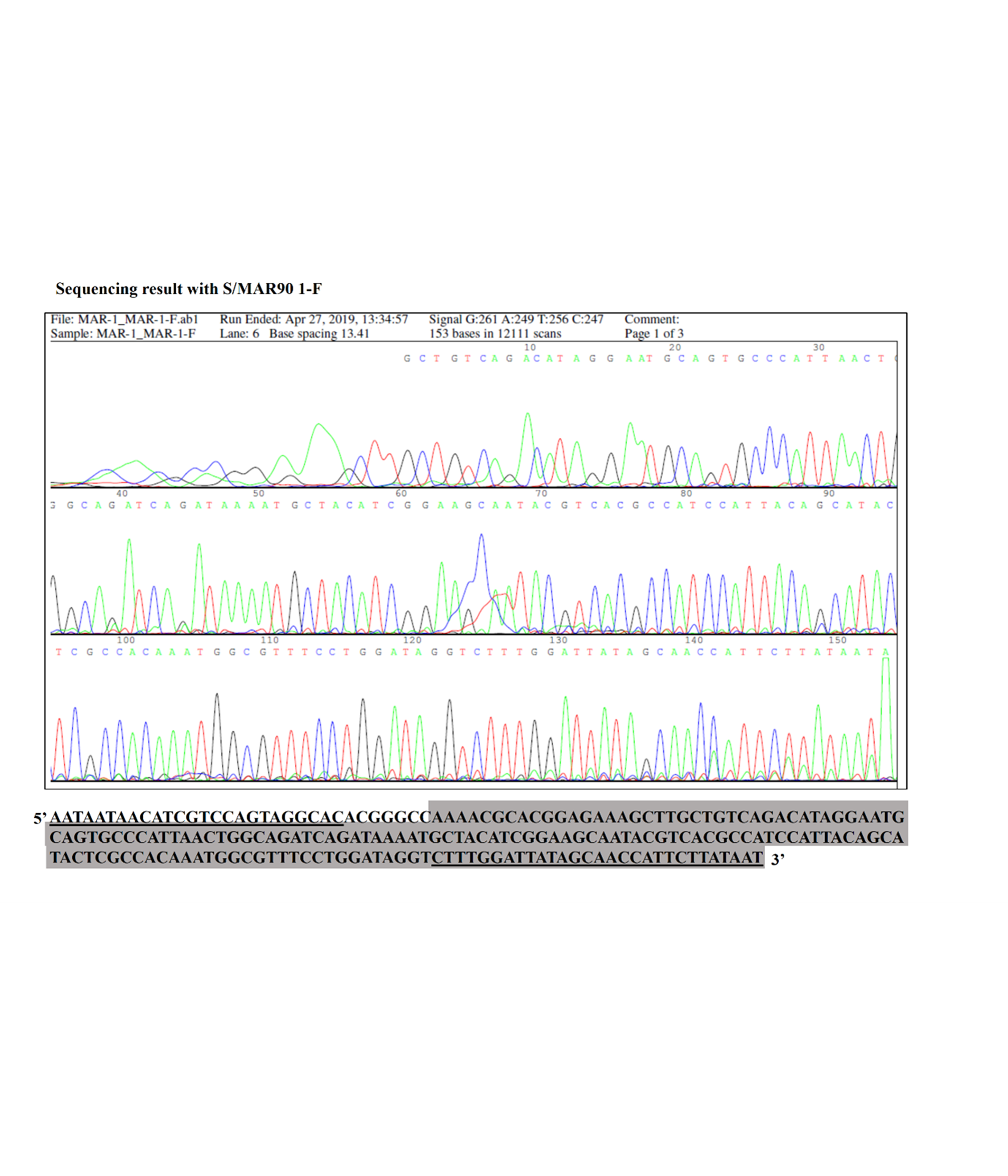

Supplement: S25 Fig — Figure shows Sanger’s DNA sequencing results with S/MAR 90 1-F primer of band excised from lane 2 (Bottom panel) in S24C Fig. (TIF) [file pntd.0009810.s025.tif]

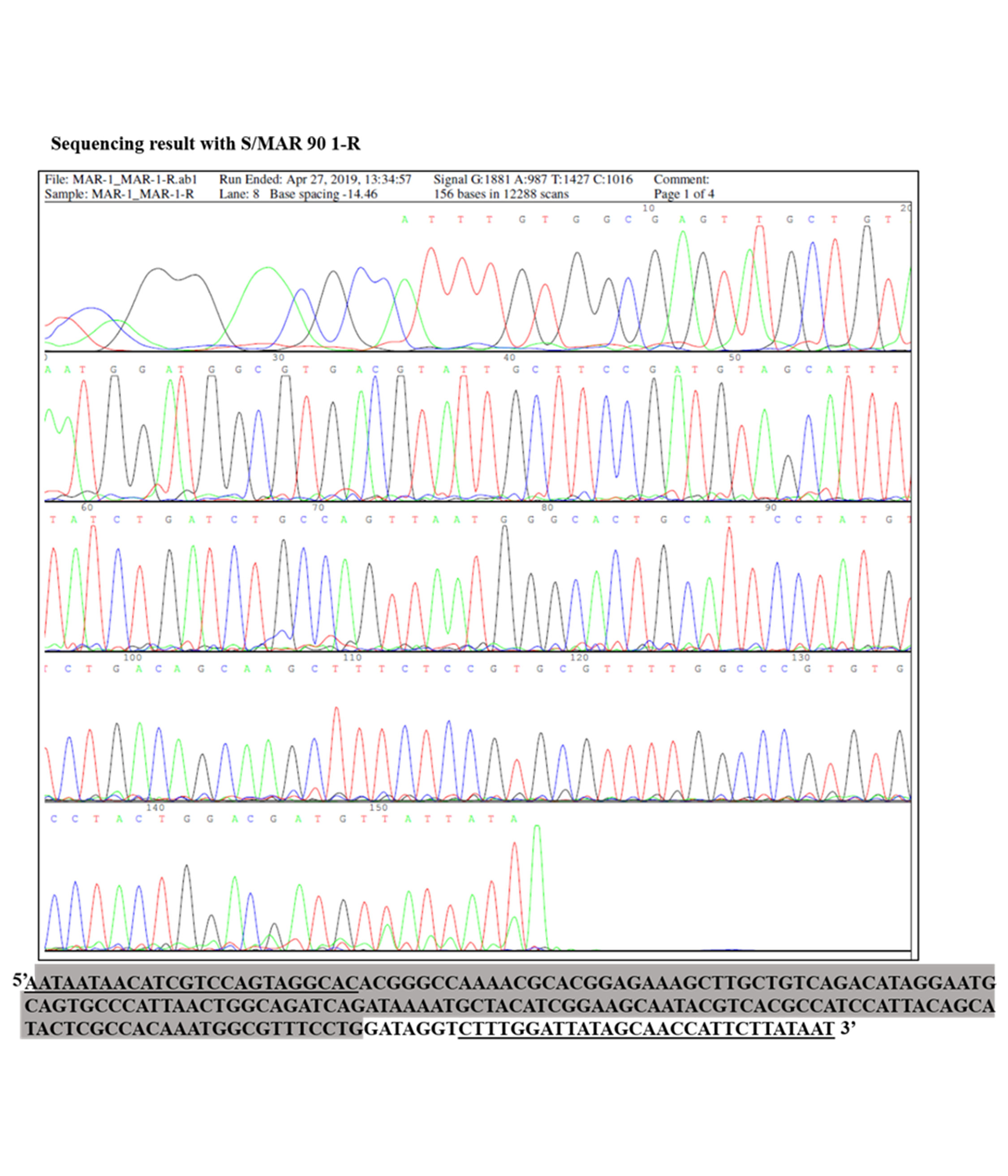

Supplement: S26 Fig — Figure shows Sanger’s DNA sequencing results with S/MAR 90 1-R primer of band excised from lane 2 (Bottom panel) in S24C Fig. (TIF) [file pntd.0009810.s026.tif]

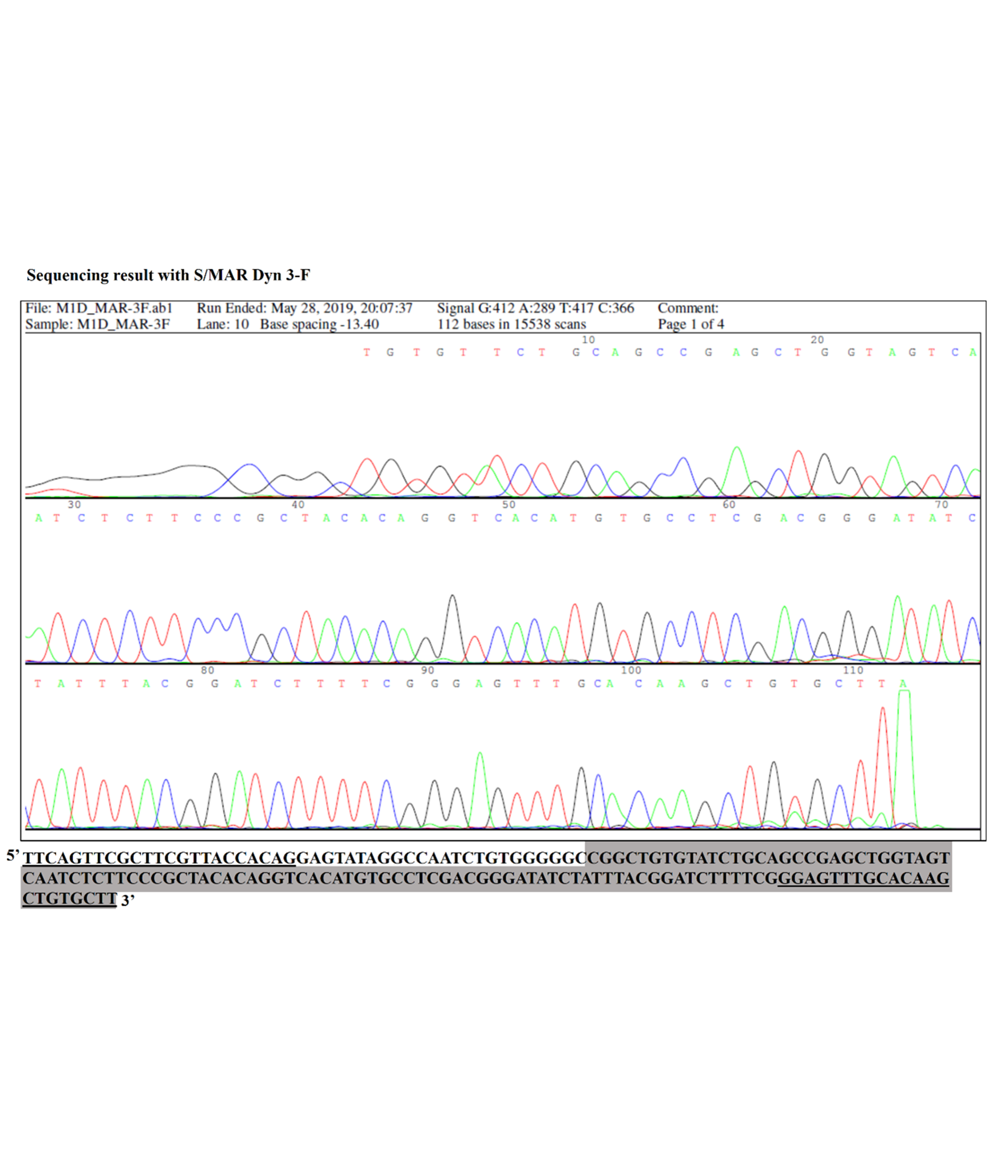

Supplement: S27 Fig — Figure shows sequencing results with S/MAR Dyn3-F primer of band excised from lane 1 (Bottom panel) in S24D Fig. (TIF) [file pntd.0009810.s027.tif]

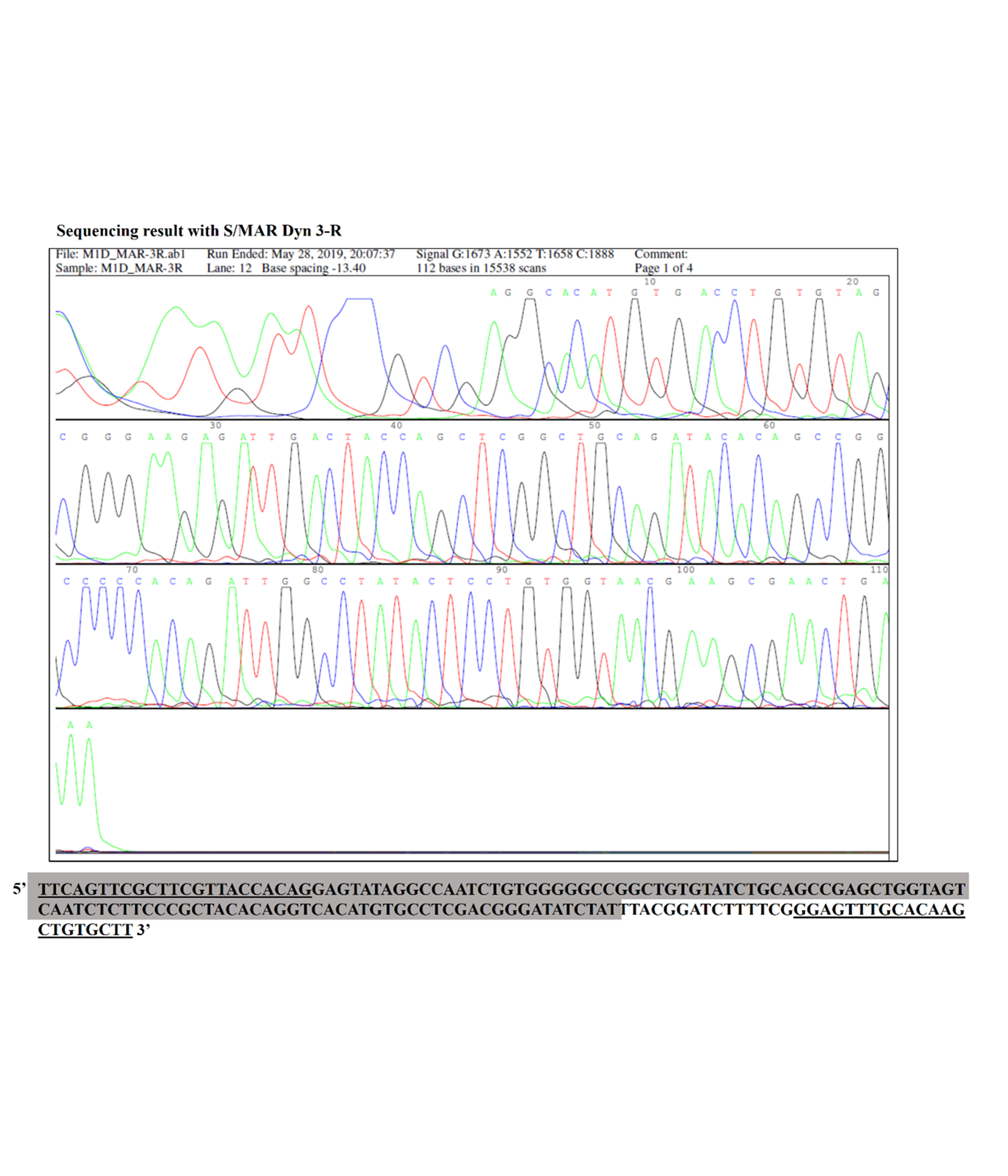

Supplement: S28 Fig — Figure shows sequencing results with S/MAR Dyn3-R primer of band excised from lane 1 (Bottom panel) in S24D Fig. (TIF) [file pntd.0009810.s028.tif]

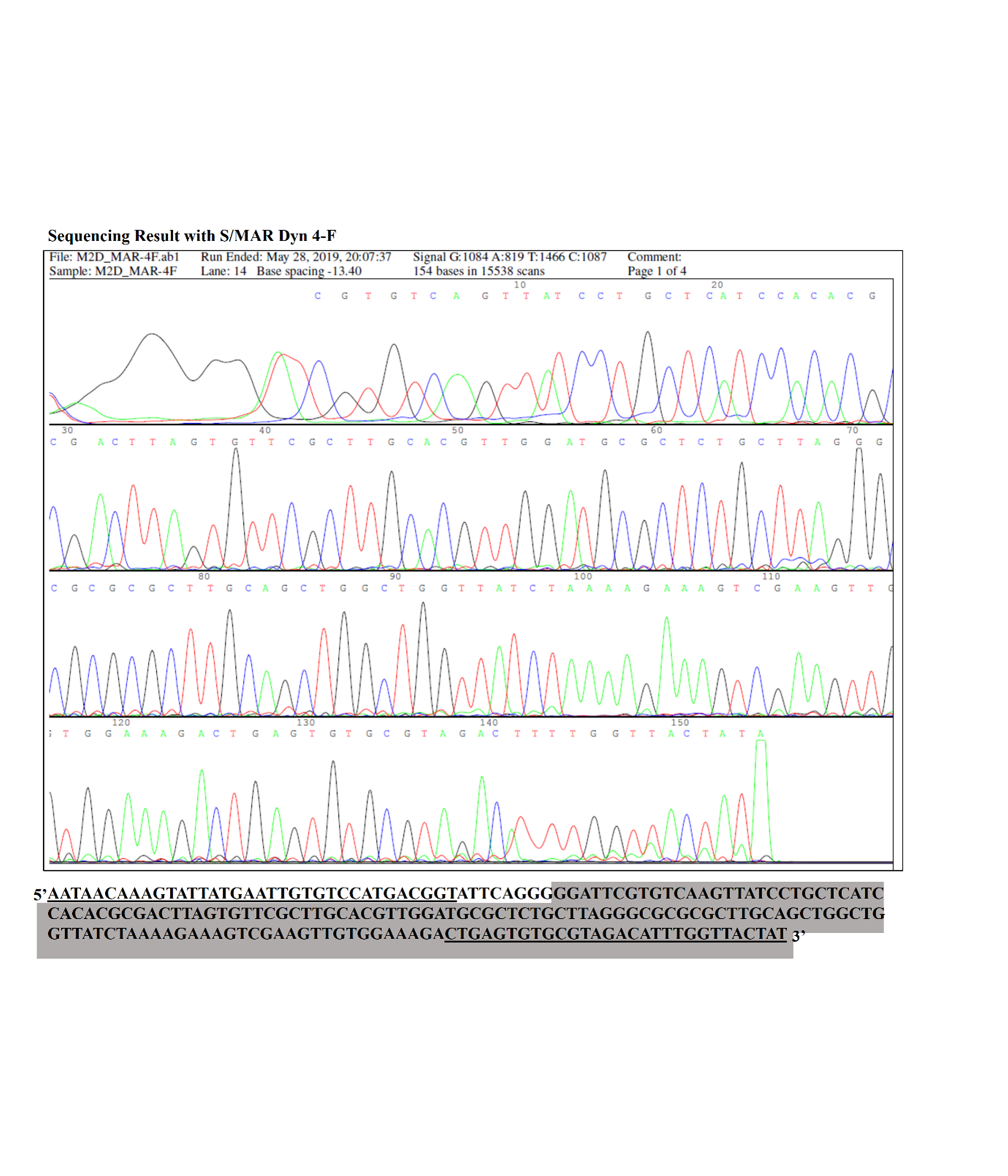

Supplement: S29 Fig — Figure shows sequencing result with S/MAR Dyn4-F primer of bands excised from lane 2 (Bottom panel) in S24D Fig. (TIF) [file pntd.0009810.s029.tif]

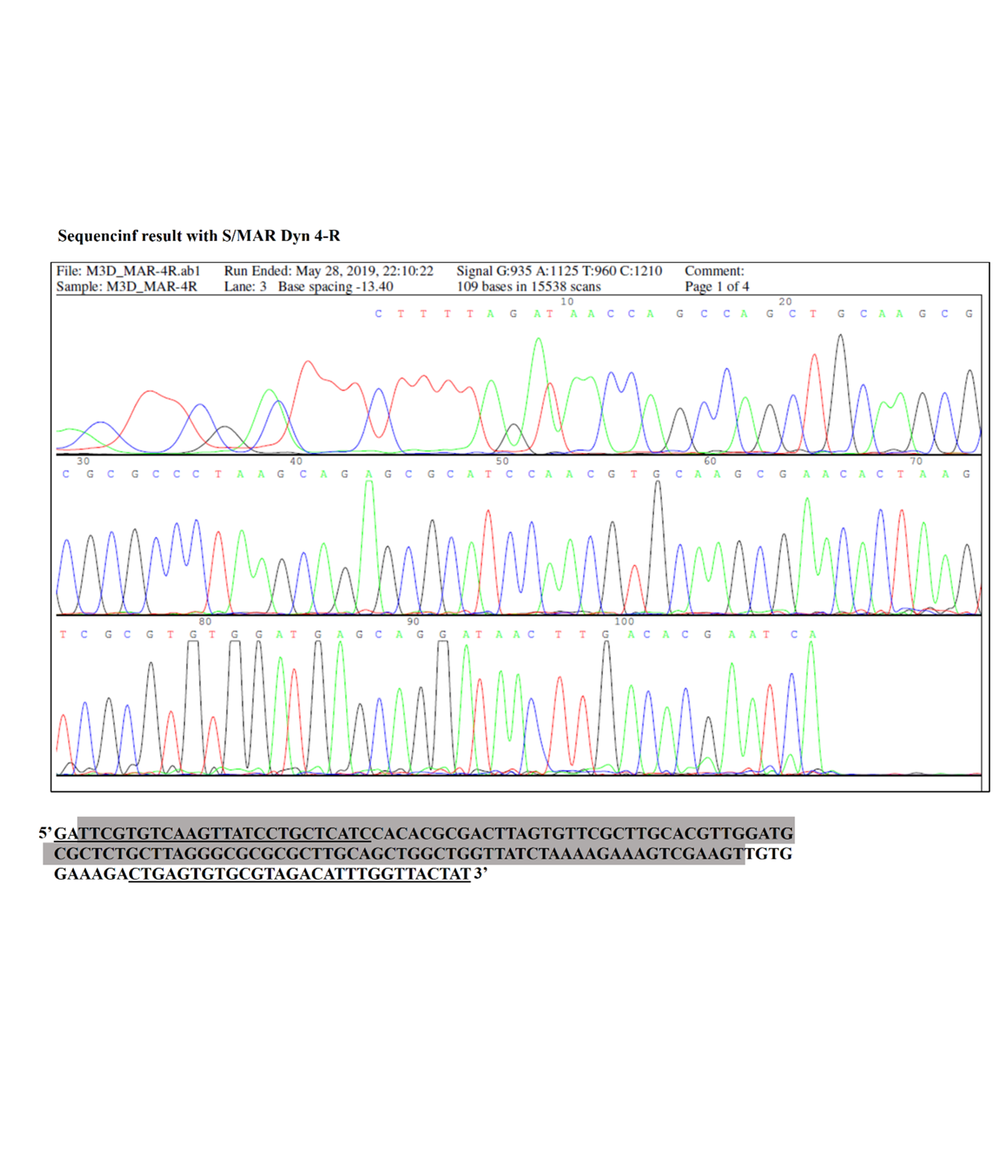

Supplement: S30 Fig — Figure shows sequencing result with S/MAR Dyn4-R primer of bands excised from lane 2 (Bottom panel) in S24D Fig. (TIF) [file pntd.0009810.s030.tif]

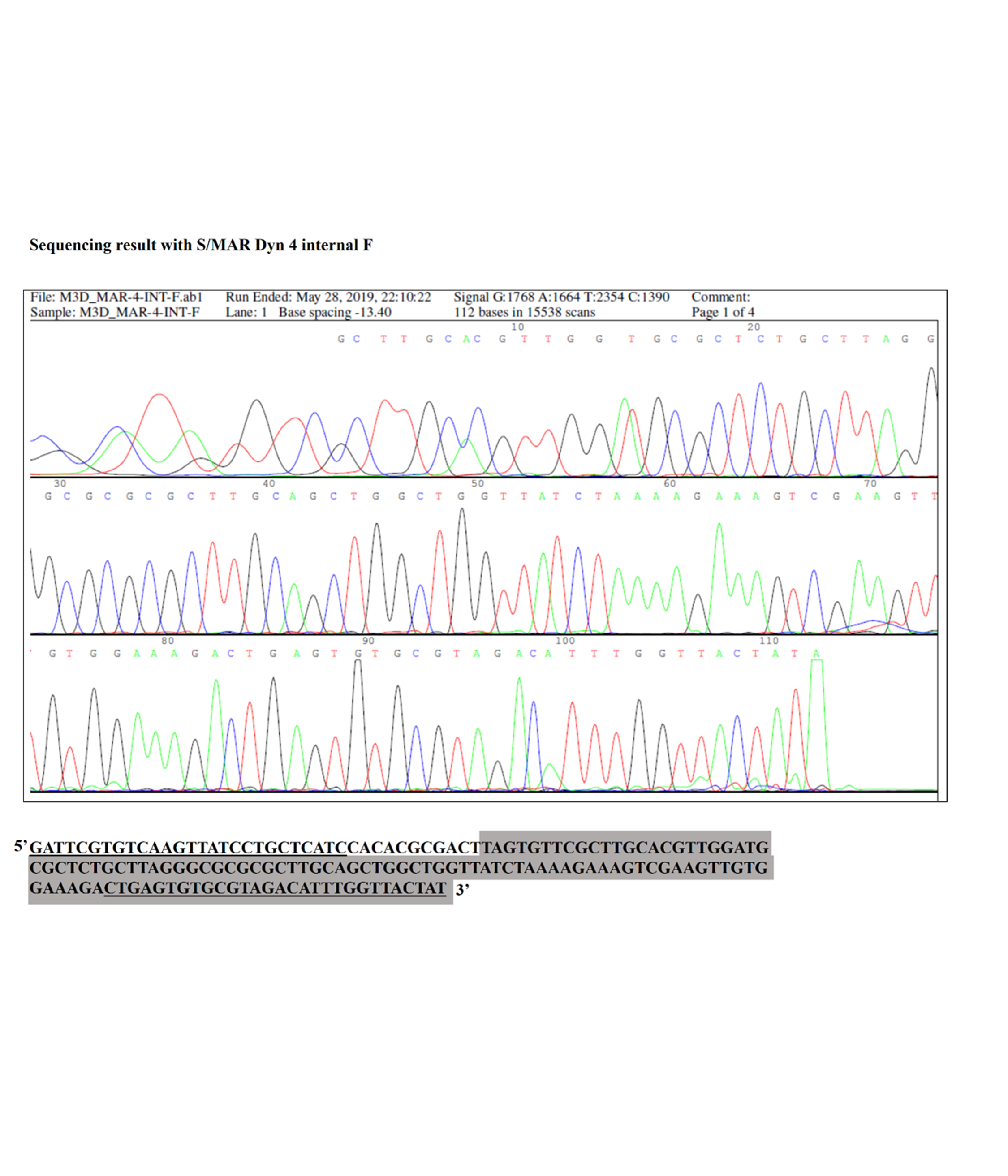

Supplement: S31 Fig — Figure shows sequencing result with S/MAR Dyn4 internal-F primer of band excised from lane 3 in S24D Fig. (TIF) [file pntd.0009810.s031.tif]

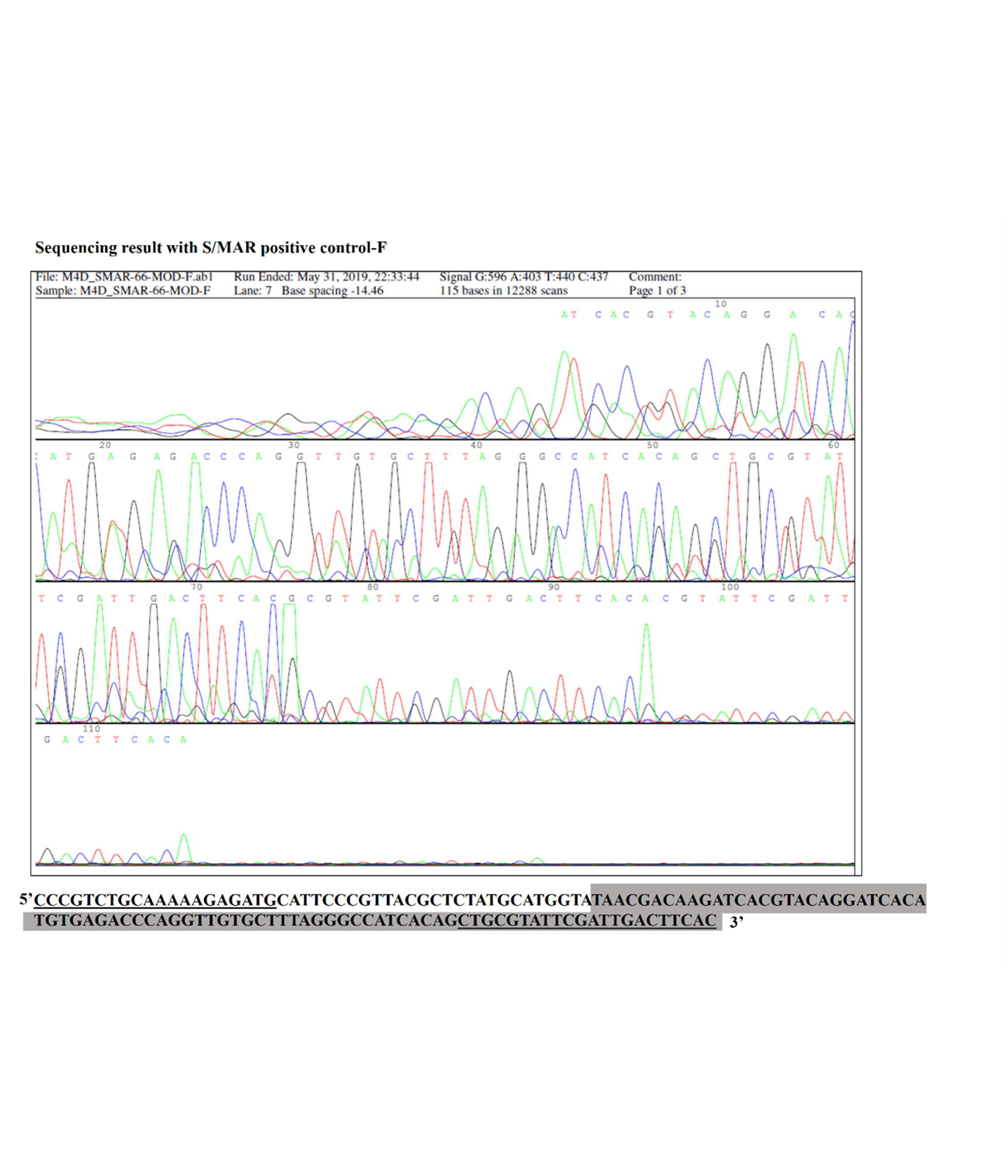

Supplement: S32 Fig — Figure shows DNA sequencing result of the positive control with S/MAR positive control-F primer. (TIF) [file pntd.0009810.s032.tif]

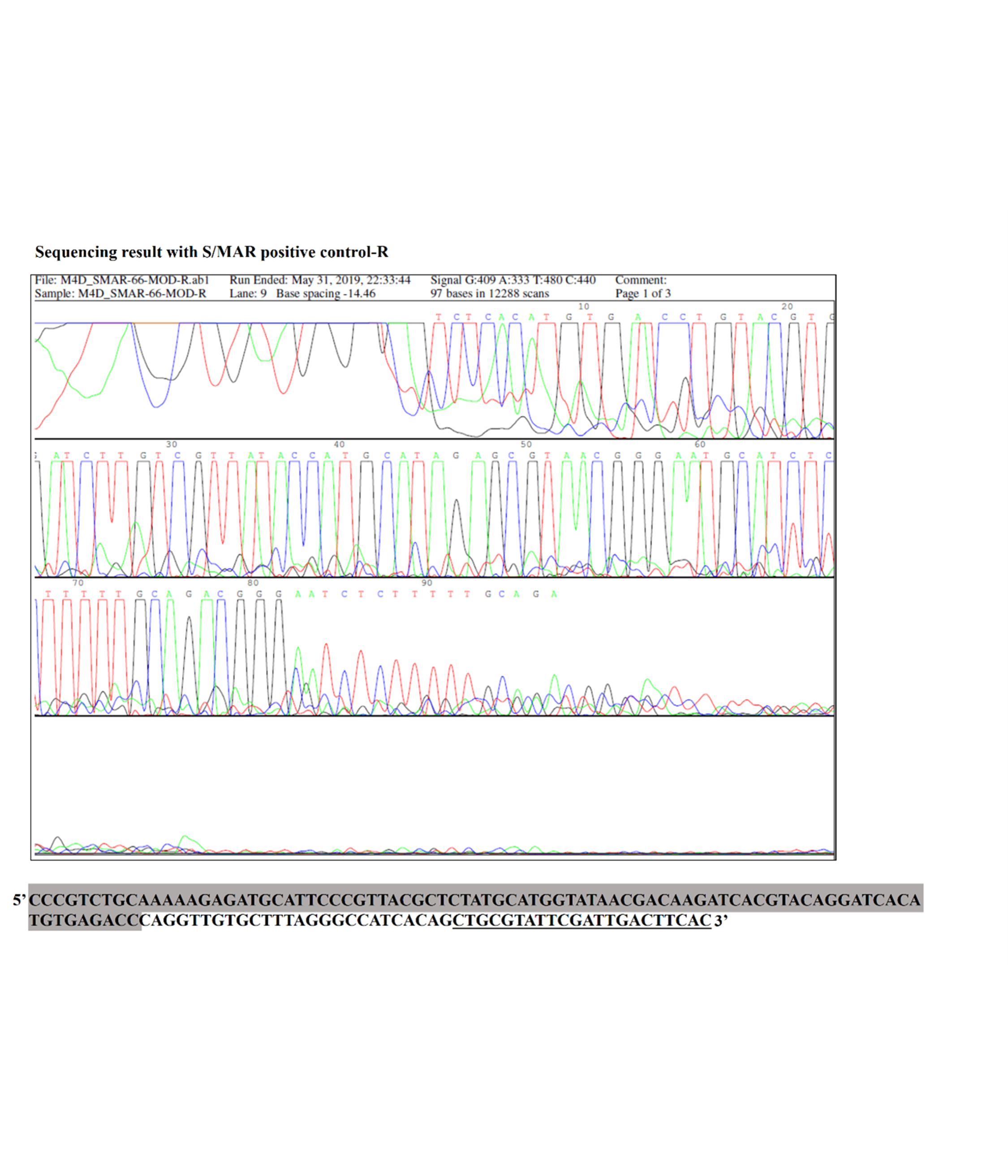

Supplement: S33 Fig — Figure shows DNA sequencing result of the positive control with S/MAR positive control-R primer. (TIF) [file pntd.0009810.s033.tif]
