# Supplementary material for: The role of nuclear organization in trans-splicing based expression of heat shock protein 90 in Giardia lamblia
Source: PLoS Negl Trop Dis. 2021 Sep 24;15(9):e0009810. doi: 10.1371/journal.pntd.0009810 (PMC8494341; doi:10.1371/journal.pntd.0009810)
Supplement: Attachment — Submitted filename: Response to reviewers_Prof. Tatu_270821.pdf [file pntd.0009810.s035.pdf]

Methods

Reviewer #1:

Concern: Statistical presentation incorrect - should show individual results when only 3 samples used. Most definitely not the SEM.

Response: As required by the reviewer, 3C results have now been shown as individual result of 3 samples. We have included the relative crosslinking frequency plots from three independent 3C experiments to show long-range interaction of HspN on chromosome 5 (Chr 5) with HspC (Chr 5), Dhcβ C-2 (Chr 5) and Dhcγ C-1 (Chr 3) in supplementary Figures S10, S13 and S16, respectively. We have also included the agarose gel images of hybrids obtained post semi-quantitative PCR in these figures.

Reviewer #2:

Concern: The objectives and hypothesis are clear and the method do address the hypothesis. However the study design still misses some silencing/knockdown experiments of the DEAD/DEXH-box RNA helicase to ensure its participation in the process.

Response: The current study predominantly focuses on biochemical, and MS based identification of proteins bound to Hsp90 pre-mRNAs. Molecular manipulations such as silencing and knock-down experiments as suggested by the reviewer will be a subject matter of our future studies.

Results

Reviewer #1:

Concern: Figures 2 and 4 are incomprehensible.

Response: To address the reviewer's point, we have made necessary modifications to simplify and improve the resolution of Figures 2 and 4. To simplify Figure 2 and 4, we have moved the quantitation data represented separately to supplementary information (Suppl. Figures S10, S13, and S16). The design and nomenclature used for different genetic loci interrogated in the vicinity and between the genes under question have also been elaborated clearly in the manuscript text (Page-14, line-365-368 in the revised article with changes highlighted) as well as Suppl. Figures S8, S9, S11, S12, S14, and S15.

Reviewer #2:

Concern: The analysis presented matches well the planned assays.

The results are clear, and some of them even elegant in design. However, since the DEAD/DEXH-box RNA helicase was isolated from a gel and the authors did not state that the corresponding area of the gels in the deleted construct were devoid of such protein, additional support for the involvement of this RNA helicase is needed.

Response: The point raised by the reviewer is well taken. We have now included the information required by the reviewer and clearly indicated the band in the HspN pre-mRNA lane in the revised version of the manuscript, which has been identified as DEAD box RNA helicase. As suggested, we have also clearly emphasized that the corresponding band from the deleted construct (HspNΔ26) did not show the presence of this RNA helicase. This clarification has now been incorporated in the result section of RNA-Protein pull down assay, page 12, line 308-310 (in the revised article with changes highlighted).

Concern: The graphical quality of the images must be improved since they appear to be prepared in a rushed manner. Image copy-pasting from the RNA folding programs is not sufficient for clarity. RNA hybrids in the splicing model should be fully written out.

Response: As suggested by the reviewer the graphical quality of the images have now been improved in the revised version of the manuscript. Images from the RNA folding programs have been removed from Figure 1D and RNA hybrids in the splicing model are now fully written out in the revised version of the manuscript.

Conclusions

Reviewer #1: Are the conclusions supported by the data presented?

Concern: Partly no, and partly I don't know because the Figures are so unclear.

Response: To make the figures clearer and more understandable, the quality as well as the resolution of figures have been improved in the revised version of the manuscript. We have also simplified Figure 2 and 4, and replaced the black and white Figures 1,2,4 and 5 with colored figures.

Concern: First, the authors do a pull-down using RNA probes, and extracts. They identify an RNA helicase, among other proteins. They claim the interaction is specific but provide no evidence that this is the case. Where are the quantitation, negative controls.

Response: Our RNA-pull down experiment is only a qualitative experiment to identify proteins interacting with the HspN pre-mRNA. We have not attempted any comparative quantitation at this stage. Also, we would like to point out that lanes 5 and 6 represent negative controls. Lane 5 represents proteins binding to Hsp90FL RNA containing only the exonic region where we do not find the protein of interest, namely RNA helicase, whereas lane 6 represents the non-specific binding of proteins to beads alone which are devoid of any RNA. This is mentioned in the results section Page-12, line- 300-302 (in the revised article with changes highlighted).

Concern: They do not provide evidence for binding of the helicase in vivo. There is also no evidence that the helicase plays any role in splicing, or even that it is in the nucleus. RNA Helicases are not generally sequence-specific and are often quite abundant. This is a very weak part of the paper and without additional functional evidence it (and all relevant Figures and tables) should be removed. As far as I can see all the authors have done is to identify a protein from a random part of a polyacrylamide gel.

Response: With all due respect, we do not think that RNA helicase identified in this study is from 'a random part of a polyacrylamide gel' as indicated by the reviewer for the following reasons-

1. The band excised for MS analysis appears reproducibly in three independent experiments of HspN RNA pull down.
2. The same band does not appear in a) beads alone control (this would be the random part of a polyacrylamide gel as indicated by the reviewer) or b) HspC RNA pull down lane.

In connection with the reviewer's comment "There is also no evidence that the helicase plays any role in splicing, or even that it is in the nucleus", we would like to clarify that helicases have often been implicated in splicing mechanisms[1-4]. In fact, the spliceosomal complex involved in facilitating RNA splicing is also known to contain RNA helicases as an integral part of the complex.

Since the time of this manuscript's submission, a study from Balan B. *et al.*,[5] reported its (RNA helicase) identification in the *Giardia* proteome and suggested its possible role in post-transcriptional regulation. Identification of this protein in our pull-down assay provides direct experimental evidence for its involvement and strengthens its possible role in post-transcriptional regulation. This manuscript has not addressed the *in vivo* binding and its role in the splicing reaction, which will be a subject matter of our subsequent manuscript.

Concern: (Figure 2) I think I vaguely understand what the authors are showing here but the Figure is really confusing and so is the Legend. What is an "EcoRI locus"? What are the numbers above and below the map? What are the products obtained with "randomly ligated" DNA? What are the "other forward primers"? What are "different loci chosen for this study"? A much clearer diagram is required showing locations and directions of all primers used. Also, to verify the results the PCR products must be sequenced.

Response: For further clarification of the Figure, we have incorporated explanations in the manuscript as well as in the Figure legends (Figure 2 and 4). The incorporated changes clarify-

1) What is an EcoRI locus?

An EcoRI locus/loci refers to the EcoRI recognition site(s) (indicated by vertical broken lines) in the vicinity and between the ORFs being investigated to determine their physical proximity in *Giardia* nucleus. The distance of the different recognition sites from the start codon of HspN has also been elaborated in Suppl. Figures S8, S11 and S14.

2) What are the numbers above and below the map?

The numbers below the schematic (-100, 0, 100, 200 series) in Figure 2A, 4A and 4D represents the precise scale of the chromosomes which indicate the position of the ORFs under investigation. In chromosome 5, the scale also indicates the position of HspN ORF relative to HspC, Dhcβ C-2 and Dhcβ C-3.

Numbers above and below the schematic (1,2,3,4,5,40,31,6,7,8,9,10,11) in Figure 2A represents the different EcoRI recognition sites chosen to investigate physical propinquity between HspN and HspC on chromosome 5.

The labels B plus 1alt, B plus 2alt, B plus 3alt, D3, D4, Calt, B, A, M2, M1, H3alt, H2alt, 1, 2 and 3 in Figure 4A represents the different EcoRI recognition sites chosen to investigate physical propinquity between HspN and Dhcβ C-2 on chromosome 5.

The labels G minus 4, G minus 3alt, G minus 1, G internal, G plus 1, G plus 2, G plus 3, G plus 4 in Figure 4D represents the different EcoRI recognition sites chosen to investigate physical proximity between HspN on chromosome 5 and Dhcɣ C-1 on chromosome 3.

3) What are the products obtained with "randomly ligated" DNA?

The products obtained with the randomly ligated DNA in the uncross-linked control gDNA panels in Figure 2B, 4B and 4E are hybrids formed using the constant primer 2F and forward primers of other EcoRI loci-

a)1F, 3F, 4F, 5F, 40F, 31altF, 6F, 7F, 8F, 9F, 10F and 11F in Figure 2B.

b) B plus 1alt-F, B plus 2alt-F, B plus 3alt-F, D3-F, D4-F, Locus Calt-F, Locus B-F, Locus A-F, M2-F, M1-F, H3alt-F, H2alt-F, 1F, 2F and 3F in Figure 4B.

c) G minus 4-F, G minus 3alt-F, G minus 1-F, G internal-F, G plus 1-F, G plus 2-F, G plus 3-F, G plus 4-F in Figure 4E.

Explanation for random ligation achieved in the case of uncross-linked control gDNA has been incorporated as Supplementary Figure S6. Random ligation is evident from the random trend of the intensities of the hybrids in the middle panel of Figure 2B, 4B and 4E.

4) What are the "other forward primers"?

"Other forward primers" refers to forward primers except for 2F (constant primer) which is constant as shown in all the lanes of Figure 2B, Figure 4B and Figure 4E.

Other forward primers are following:

a)1F, 3F, 4F, 5F, 40F, 31altF, 6F, 7F, 8F, 9F, 10F and 11F in Figure 2B.

b) B plus 1alt-F, B plus 2alt-F, B plus 3alt-F, D3-F, D4-F, Locus Calt-F, Locus B-F, Locus A-F, M2-F, M1-F, H3alt-F, H2alt-F, 1F, 2F and 3F in Figure 4B.

c) G minus 4-F, G minus 3alt-F, G minus 1-F, G internal-F, G plus 1-F, G plus 2-F, G plus 3-F, G plus 4-F in Figure 4E.

5) What are "different loci chosen for this study"?

"The different loci chosen for this study" refers to the EcoRI recognition sites represented as broken vertical lines in the schematics in Figure 2A, Figure 4A and 4D.

Detailed explanations for the above points have also been incorporated in the Supplementary Figures S6, S7, S8, S9, S11, S12, S14 and S15, indicated explicitly in the revised manuscript text. Figures 2 and 4 have also been simplified to convey the results more clearly.

As suggested by the reviewer a much clearer diagram showing the location and directions of all primers used in 3C has been illustrated in the revised supplementary information (Suppl. Figures S9, S12 and S15). The sequencing result of the enriched hybrid in each 3C experiment have now been included in the main Figures 2C and 4C and 4F.

Concern: In panel C of Figure 2, with only 3 measurements you cannot legitimately use the standard error of the mean or even the standard deviation. Please show the individual measurements instead.

Response: As suggested by the reviewer we have now modified the previous representation of crosslinking frequency to include the graphs for individual measurements instead. The relative crosslinking frequency showing interaction between 1) HspN with HspC, 2) HspN with Dhcβ C-2 and 3) HspN with Dhcɣ C-1 have now been shown individually in supplementary information (Suppl. Figures S10, S13 and S16) along with the agarose gel images after semi-quantitative PCR.

Concern (Figure 3): This shows that probes that hybridize to HspN and HspC ORFs colocalize. That is interesting, but I am not sure about the negative control gene. Is this gene on the same chromosome? If not, it is not valid as a control. A better control would be to try two different genes that are located in between the HspN and HspC ORFs on the same chromosome. Another

control that is essential is to do the same experiment after inhibiting transcription, to see whether the mutual association depends on RNA.

Response: The query raised by the reviewer regarding negative control gene being on the same chromosome has been addressed in the revised version. We have now performed DNA-FISH for two different genes that are located in between the HspN and HspC ORFs on the same chromosome to serve as a negative control. We observe that the two different genes (Enolase gene- GL50803_11118 and Protein 21.1- GL50803_24412) between HspN and HspC on the same chromosome do not colocalize and therefore now serves as true negative control for our DNA-FISH experiment. The different panels from this experiment have now been incorporated in Figure 3.

The suggestion made by the reviewer is relevant and we would pursue such regulatory aspects of the long-range interaction in our subsequent manuscript that will interrogate the spatio-temporal localization of different pre-mRNAs from split genes.

Concern: Figure 4 is truly incomprehensible. I gave up trying to understand it. If the conclusion is that the two separate *trans*-spliced loci are all together in one place, this actually seems strange because I would not expect it. (This is not the equivalent of spliced leader *trans*-splicing, and most trypanosomes genes are also not located near the spliced leader array.) In any case, the results need to be be confirmed by sequencing and by in situ hybridisation, again with additional appropriate controls.

Any discussion is premature because this needs more experimental work in order to support the conclusions.

Response: To make Figure 4 more understandable, clarification about the different loci used for the study has been incorporated in the text of revised manuscript. The map showing the linear genomic distance between HspN and Dhcβ C-2 on the same chromosome has been shown and the direction of the primers used for the study has been clearly indicated in supplementary information (Suppl. Figure S11 and S12). The map showing the relative distances between the chosen EcoRI loci on chromosome 3 along with the direction of flanking primers to examine physical proximity between HspN on chromosome 5 and Dhcɣ C-1 on chromosome 3, have been elaborated in supplementary information (Suppl. Figure S14 and S15).

Though agreeing with the fact that this *trans*-splicing process is very different from SL-*trans*-splicing, we stand by our findings from 3C experiments that suggest nuclear proximity of *trans*-spliced genes. It actually makes perfect sense that the genes coding for the *trans*-spliced exons be in proximity to facilitate juxta positioning of corresponding pre-mRNAs to expedite the *trans*-splicing reaction. However, to address the reviewer's queries, we have further performed hybrid sequencing and in situ hybridization with appropriate controls. Our experiments clearly bear out that the *trans*-spliced genes are in proximity within the nucleus.

The sequencing result of the enriched hybrids in each 3C experiment to investigate the physical proximity of HspN with HspC, Dhcβ C-2 and Dhcɣ C-1 has been included in the manuscript Figures 2 and 4. Additionally, the DNA FISH result validating the interaction between *trans*-spliced genes-HspN (on chromosome 5) with the dynein genes, Dhcβ C-2 (chromosome 5), Dhcβ C-3 (chromosome 5) and Dhcɣ C-1(chromosome 3) along with the appropriate negative control has now been incorporated in the revised version of the manuscript as Figure 5.

Reviewer #2:

Concern: For the most part, conclusions are supported, but additional support for the DEAD/DEXH-box RNA helicase is needed.

Response: Our results demonstrating binding of RBPs to HspN pre-mRNA through RNA-protein pull down followed by MS based identification are convincing. We have also shown that RNA helicase selectively interacts with HspN pre-mRNA and not seen in the controls. Understanding detailed role and mechanism by which the putative helicase may facilitate Hsp90 *trans*-splicing will be the subject matter of our future studies.

Concern: As stated before, the limitations are not clearly described on this issue alone.

Response: As suggested by the reviewer, limitations concerning the results pertaining to RNA helicase have now been incorporated in the revised version of the manuscript (Page-13, Lines 337-339- in the revised article with changes highlighted). These include- 1) the possibility that the RNA helicase may be of general nature, also interacting with other RNAs in the nucleus. 2) further biochemical experiments are required to understand the mechanistic aspects of this protein.

Concern: The authors do discuss the importance and relevance of their study, including public health issues.

Response: As per the reviewer's suggestion, the importance and relevance of this study, including public health issues – "Our attempt to understand the *in vivo* mechanism of Hsp90 *trans*-splicing could indeed be useful to further expand our understanding of the molecular mechanisms of this elegant process. In addition, the results from our study could be leveraged to design better anti-giardial drugs to combat *Giardiasis*, which remains to be a public health concern in developing and under-developed countries." is now discussed in the study, on page-22, line-588-592 (in the revised article with changes highlighted).

Editorial and Data Presentation Modifications

Reviewer #1:

Concern: The English needs correction, articles ("The" and 'a") are pervasively missing.

Lines 72-74 - why do some species have capital letters and others not? None should, and trans splicing doesn't need a capital letter either. Trans should be italicised, though.

Line 323 - "dispersed" is the wrong word. it should be "separated".

Accession numbers for sequences not generated in the paper can be mentioned in the text but so not need to be provided in a separate section.

Response: The reviewer's suggestions have been incorporated in the lines indicated. "Trans" in *trans*-splicing has been italicized throughout the manuscript text.

The separate section for accession numbers of sequences has been removed and the same has been indicated in the text of the revised version of the manuscript along with the corresponding genes upon their first mention in the text of the 3C results.

Reviewer #2:

Concern: In the introduction, reference 12 is not the best choice for the statement: Sequence analysis of HspN and HspC pre-mRNA substrates showed the presence of cis-sequence elements; GU-AG intron-exon boundary elements, polypyrimidine tract as well as branch point adenine in Hsp90 pre-mRNAs.

Response: As suggested by the reviewer we have replaced reference 12 with more appropriate references (PMIDs-9016643, 23074130) in the manuscript on Page-4, line-89 (in the revised article with changes highlighted).

Summary and General Comments

Reviewer #1:

Concern: If the colocalization is true it would be interesting, but more experimental data are needed.

Response: We thank the reviewer for this concern. As suggested by the reviewer, we have included DNA-FISH results of two genes between HspN and HspC, serving as a true negative control to strengthen our observation of colocalized HspN and HspC genes in the *Giardia* nucleus. To provide additional confirmation of our 3C results, we have carried out DNA- FISH with other

trans-spliced genes as well, and results of the same have now been incorporated in the manuscript as Figure 5.

Reviewer #2: This study is highly relevant to the field of co-transcriptional splicing in *Giardia*. The authors cleverly used 3C assays to understand *in vivo* transcription and *trans*-splicing of the Hsp90 separate transcripts with the aid of an RNA helicase. This work provides the first glimpse to nuclear events in *Giardia* with therapeutic potential.

Response: We thank the reviewer for this motivation.

References

1. Will, C.L. and R. Lührmann, *Spliceosome structure and function.* Cold Spring Harb Perspect Biol, 2011. **3**(7).
2. Cordin, O. and J.D. Beggs, *RNA helicases in splicing.* RNA Biol, 2013. **10**(1): p. 83-95.
3. Liu, Y.-C. and S.-C. Cheng, *Functional roles of DExD/H-box RNA helicases in Pre-mRNA splicing.* Journal of Biomedical Science, 2015. **22**(1): p. 54.
4. Strittmatter, L.M., et al., *psiCLIP reveals dynamic RNA binding by DEAH-box helicases before and after exon ligation.* Nature Communications, 2021. **12**(1): p. 1488.
5. Balan, B., et al., *Multimodal regulation of encystation in Giardia duodenalis revealed by deep proteomics.* Int J Parasitol, 2021.
